# Plan Quality and Secondary Cancer Risk Assessment in Patients with Benign Intracranial Lesions after Radiosurgery using the CyberKnife M6 Robotic Radiosurgery System

**DOI:** 10.1038/s41598-019-46133-8

**Published:** 2019-07-09

**Authors:** Jen-Hong Lan, Chin-Shiuh Shieh, Chao-Hong Liu, I-Chun Cho, I-Hsing Tsai, Long-Chun Chen, Pei-Ju Chao, Hsiao-Fei Lee, Yu-Jie Huang, Tsair-Fwu Lee

**Affiliations:** 1 Medical Physics and Informatics Laboratory of Electronics Engineering, National Kaohsiung University of Science and Technology, Kaohsiung, Taiwan Republic of China; 2 Department of Radiation Oncology, Kaohsiung Chang Gung Memorial Hospital and Chang Gung University College of Medicine, Kaohsiung, Taiwan Republic of China; 3 Department of Dermatology, Kaohsiung Yuan’s General Hospital, Kaohsiung, Taiwan Republic of China; 4 PhD program in Biomedical Engineering, Kaohsiung Medical University, Kaohsiung, Taiwan Republic of China

**Keywords:** Risk factors, Radiotherapy, Risk factors, Radiotherapy

## Abstract

This study was performed to examine the quality of planning and treatment modality using a CyberKnife (CK) robotic radiosurgery system with multileaf collimator (MLC)-based plans and IRIS (variable aperture collimator system)-based plans in relation to the dose–response of secondary cancer risk (SCR) in patients with benign intracranial tumors. The study population consisted of 15 patients with benign intracranial lesions after curative treatment using a CyberKnife M6 robotic radiosurgery system. Each patient had a single tumor with a median volume of 6.43 cm^3^ (range, 0.33–29.72 cm^3^). The IRIS-based plan quality and MLC-based plan quality were evaluated by comparing the dosimetric indices, taking into account the planning target volume (PTV) coverage, the conformity index (CI), and the dose gradient (R_10%_ and R_50%_). The dose–response SCR with sarcoma/carcinoma induction was calculated using the concept of the organ equivalent dose (OED). Analyses of sarcoma/carcinoma induction were performed using excess absolute risk (EAR) and various OED models of dose–response type/lifetime attributable risk (LAR). Moreover, analyses were performed using the BEIR VII model. PTV coverage using both IRIS-based plans and MLC-based plans was identical, although the CI values obtained using the MLC-based plans showed greater statistical significance. In comparison with the IRIS-based plans, the MLC-based plans showed better dose falloff for R_10%_ and R_50%_ evaluation. The estimated difference between Schneider’s model and BEIR VII in linear-no-threshold (Lnt) cumulative EAR was about twofold. The average values of LAR/EAR for carcinoma, for the IRIS-based plans, were 25% higher than those for the MLC-based plans using four SCR models; for sarcoma, they were 15% better in Schneider’s SCR models. MLC-based plans showed slightly better conformity, dose gradients, and SCR reduction. There was a slight increase in SCR with IRIS-based plans in comparison with MLC-based plans. EAR analyses did not show any significant difference between PTV and brainstem analyses, regardless of the tumor volume. Nevertheless, an increase in target volume led to an increase in the probability of SCR. EAR showed statistically significant differences in the soft tissue according to tumor volume (1–10 cc and ≥10 cc).

## Introduction

Stereotactic radiosurgery (SRS) is either a key treatment choice to achieve a cure or an additional treatment option to surgical resection for the treatment of patients with benign intracranial tumors or brain metastases. There has been a considerable increase in the application of SRS due to its relatively weak side effects and good rate of local control^1,2^. In our department, intracranial lesions are treated using a multileaf collimator (MLC) or an IRIS variable aperture collimator system. These methods are based on use of the CyberKnife M6 system (Accuray Inc., Sunnyvale, CA) with a pair of orthogonal kV X-ray imaging systems with simultaneous target tracing^3^.

Treatment of patients with intracranial tumors using SRS has been shown to confer benefits for both survival and quality of life^4^. Nevertheless, SRS is still applied with caution in younger patients due to concerns regarding secondary cancer risk (SCR) in such cases. It is essential to evaluate the quality of treatment plans and SCR for healthy structures in which the tolerance to radiation is low. The peripheral dose (PD) received outside the field of therapeutic radiation can result in SCR. The PD is composed of scatter radiation from a number of sources, i.e., leakage from the treatment machine head and the collimators. The PD scatter contributions are affected by the characteristic features of the treatment plan, in particular the type of collimator, monitor unit (MU), aperture size, beam number and orientations, etc.^5,6^. Delaby *et al*.^5^ reported that the evaluation of PD received by healthy tissues in brain stereotactic radiotherapy treatment (SRT) using a CyberKnife M6 system demonstrated that PD was approximately 0.06% of MU with a 20-mm fixed collimator and 0.04% of MU with the identical aperture for an IRIS collimator^5^. Although MLC-based plans were not included in their study, the results are unlikely to be markedly different. Patient scatter is another source of PD. Although PD and MU are different concepts, the level of PD should be directly related to the MU plan, with higher MU plans having more leakage and greater scatter and therefore higher PD. The old and new versions of the CyberKnife M6 system differ due to the use of newer IRIS and MLC collimator systems. It is necessary to investigate the SCR and the quality of CK treatment plans. However, there have been no studies using the latest version of the system in CK MLC/IRIS-based plans taking into account dosimetric plan quality and dose–response SCR analyses in patients with benign intracranial tumors. However, a major uncertainty of the SCR estimates is the lack of data regarding the shape of the dose–response relationship; scenarios in different dose–response SCR models should be evaluated.

## Materials and Methods

### Patients

The study population consisted of 15 patients with benign intracranial tumors under curative treatment using CyberKnife M6. Each patients underwent treatment with the IRIS technique using the CyberKnife M6 system or an InCise MLC. The characteristics of the patients are presented in Table 1. Overall, the 15 patients enrolled in the study had a single tumor with a median volume of 6.43 cm^3^ (range, 0.33–29.72 cm^3^). The mean age of the patients was 54 years (range, 15–67 years). To investigate the age dependence of SCR, patients of different ages at the time of treatment were selected. All experimental protocols of this study were approved by the institutional review board (IRB) of Chang Gung Memorial Hospital. Due to this a retrospective study, the requirement for informed consent was waived in accordance with local laws and regulations (IRB approval No. 201701596B0 and 201802377B0). All of the studies and experiments were performed in accordance with specific regulations, rules, and guidelines.

**Table 1:** Patient characteristics and dose conversion.

Patients	Age at exposure	Tumor type	Tumor volume (cm³)	Prescribed Dose (Gy)
1	15	AVM	6.43	18
2	22	AVM	14.29	16
3	23	AVM	6.40	16
4	35	Dural AVFs	0.33	12
5	39	Dural AVFs	4.11	15
6	43	Dural AVFs	5.45	18
7	53	Dural AVFs	7.95	15
8	54	AVF	27.39	14
9	57	Dural AVFs	26.10	15
10	58	Residual Meningioma	14.93	18
11	59	AVM	29.72	16
12	64	Dural AVFS	7.28	17
13	64	AVF	2.32	16
14	66	LT IAC	0.99	12
15	67	LT IAC	0.82	12

Notes: BED conversion factors used for PTV were α/β = 10 Gy; those for OARs were α/β = 3 Gy for 2 Gy/fraction, respectively. Patients are listed according to age from youngest to oldest.

Abbreviations: M, Male; F, Female; AVM, Arteriovenous malformation; Dural AVFs, Dural Arteriovenous Fistula; AVF, Arteriovenous Fistula; LT IAC, Left Intracranial Arachnoid Cyst; IRIS, Iris collimator; BED, Biologically Effective Dose.

### Treatment planning

Treatment plans were created using Multiplan Treatment Planning Software (MTPS; version 5.1.3; Accuray Inc., Chesapeake Terrace, Sunnyvale, CA). A LightSpeed RT16 CT simulator was used to scan the patients at a slice thickness of 0.625 mm comprised of 512 × 512 pixels at dimensions of 0.9375 × 0.9375 × 0.625 mm^3^ (GE Medical Systems, Milwaukee, WI). Fusion of the CT images was completed with images obtained by magnetic resonance imaging (MRI) with a slice thickness of 1.0 mm (GE Signa Excite MR Scanner; GE Medical Systems) to delineate the planning target volume (PTV). Patients were treated using plans based on either MLC or IRIS, and comparisons were made with additional re-based planning. In each case, identification and verification of the organs at risk (OAR) and PTV was performed by one radiation oncologist. The MLC/IRIS-based plans implied use of the same OAR dose constraints and prescribed doses.

The PTV provided for an additional three-dimensional 1.0-mm safe margin of gross tumor volume (GTV) to take into account motion uncertainties and setup problems of the patients. The prescribed isodose line (IDL) was 80%, and the prescribed doses were 12–18 Gy (median, 16 Gy). The planning goal for the PTV was application of a minimum dose to >95% of the target. The range of PTV in all 15 patients was 0.33–29.72 cm^3^ with a median of 6.43 cm^3^. The given penalties aimed to ensure proper management of the PTV dose and to spare the OAR in case the tumor did not receive a sufficient dose, as prescribed, and the constrained doses were lower than the actual OAR. The following biologically effective dose conversion factors for 2 Gy/fractions were used: α/β = 10 Gy for PTV; α/β = 3 Gy for OAR^2,7^.

### Dosimetric evaluation

Plan quality assessment consisted of dosimetric indices, i.e., PTV coverage and a conformity index (CI). Evaluation of PTV coverage was performed using the following equation: coverage = target isodose volume (TIV)/PTV. Evaluation of CI was performed using the equation: CI = prescribed isodose volume (PIV)/TIV.

The performances of the MLC- and IRIS-based plans were evaluated using the dose gradient (R_50%_) to check whether to form a deep dose gradient; the R_50%_ is viewed as the ratio of the volume covered by the 50% prescribed isodose volume of the maximum target dose (D_50%_) to the PTV^8–10^. R_10%_ was computed to assess the dose falloff to the region of low dose; the ratio of R_10%_ is the ratio of the volume encompassed by the 10% prescribed isodose volume of the maximum target dose (D_10%_) to the PTV^8–10^.

### Secondary cancer risk assessment

SCR assessment was performed after extraction of dose–volume histograms (DVHs) from the MTPS. Measurement of the non-homogeneous type organ dose distributions in the area of high dose was performed using the organ equivalent dose (OED) concept^11^, as described and used in previous studies^12–14^. The inductions of carcinomas and sarcomas were evaluated and modeled separately.

### Analyses of carcinoma induction

SCR has a specific phenomenological modeling concept implying use of OED as suggested by Schneider *et al*.^11^. The effects of repopulation and proliferation as well as cell killing were taken into consideration and the required data were fitted to models of OED using the full models of parameterization, plateau dose–response, linear–exponential, and linear types. In the present study, the above-mentioned EAR and OED dose–response models were used for analysis of carcinoma induction. All relevant details were described previously^7,11,14,15^, and a brief description is presented below.

Risk equivalent dose (RED) can be defined as a tissue dose value with a dose–response relationship that is a function of the SCRs. RED is considered to be a weighting average over the OED; the value is a function of cancer risk induction for the OARs and is expressed in Gy. Considering the fractionation effect, given by *a*′ = *a* + *βD*, *a*/*β* = 3*Gy* was used for the OARs. The EAR was also estimated using RED as described previously^14,16,17^. Thus, when exposed to RED at one age (age_x,_ initial age) with re-exposure at an older age (age_a,_ older age), the EAR can be defined as the risk in an organ with volume V_i_ where parameters *γ*_*e*_ and *γ*_*a*_ are the age modifying factors and β is defined for people exposed at age 30 years and again at age 75 years^17,18^.

The OED for carcinoma induction (Eq. 1) was used to estimate the risk of radiation-induced secondary cancer of the brainstem.

1 $$OE{D}_{c}=\frac{1}{V}{\sum }_{i}Vi\cdot \,\frac{{e}^{-{a}_{i}{\rm{^{\prime} }}{D}_{i}}}{{a}_{i}{\rm{^{\prime} }}R}\,(1-2R+\,{R}^{2}{e}^{{a}_{i}{\rm{^{\prime} }}{D}_{i}}-{(1-R)}^{2}{e}^{-\frac{{a}_{i}{\rm{^{\prime} }}R}{1-R}{D}_{i}}$$

### Analyses of sarcoma induction

Investigation of the SCR in terms of PTV and soft tissue was performed using the sarcoma induction model (Eq. 2), which makes it possible to obtain OED^17^.

The applied parameters included total dose (D), repopulation (R), dose per fraction (dF), and cell killing parameter (α).

2 $$OE{D}_{s}=\frac{1}{V}{\sum }_{i}Vi\cdot \,\frac{{e}^{-{a}_{i}{\rm{^{\prime} }}{D}_{i}}}{{a}_{i}{\rm{^{\prime} }}R}\,(1-2R+\,{R}^{2}{e}^{{a}_{i}{\rm{^{\prime} }}{D}_{i}}-{a}_{i}{\rm{^{\prime} }}R{D}_{i}-{(1-R)}^{2}{e}^{-\frac{{a}_{i}{\rm{^{\prime} }}R}{1-R}{D}_{i}}$$

3 $$a^{\prime} =a+\beta {D}_{i}\cdot \frac{{d}_{F}}{D}$$

OED is viewed as the sum of voxels (values of RED) divided by the number of voxels (N), with the total volume of organs, V. Supplementary Table S1 shows all of the parameters used for both the EAR models and Schneider’s OED. The values of R, γ_e_, γ_a_, and β parameters in excess cases per 10,000 person-years (PY) were determined from previous studies^17,19^.

In the present study, we conducted analyses of sarcoma induction using four OED dose–response models and EAR models, i.e., intermediate repopulation (SI_-R0.5_), linear-no-threshold (Lnt), full tissue recovery (SF_-R1.0_), and low repopulation (SL_-R0.1_). Analyses of sarcoma induction were performed using different dose–response models taking into account the different effects of repair/repopulation using Eq. 2 and the respective limitation of the fixed R = 0.1, R = 0.5, R = 1.0^17,18,20^.

### BEIR VII model

Evaluation of the SCR for induction of sarcoma and carcinoma was also performed using the BEIR VII model. The low dose was defined as the average dose absorbed by the organ, as cancer induction has a dose–response type relationship and can be presented using a linear dose function. The described model derived the Lnt model from the Hiroshima atomic bomb survivors, and the obtained data can be combined with the data regarding cancer risk with irradiation dose (approximately 40 Gy)^17^.

### Evaluation of EAR

The EAR (Eq. 4) is represented as a function of OED multiplied by the initial slope of the corresponding dose–response curve (low-dose region)^21^. The EAR has units of excess cases per 10000 person-years (PYs)/Gy.

4 $$EAR(D,e,a,s)=OED\cdot \beta \cdot {e}^{({\gamma }_{e}[e-30]+{\gamma }_{a}\mathrm{ln}[\frac{a}{75}])}(1\pm {\rm{s}})$$

### Evaluation of lifetime attributable risk

Lifetime attributable risk (LAR), defined as the lifetime likelihood (percentage) of developing secondary cancer (baseline risk time), was evaluated using Eq. 5, taking into consideration the age of the patient at the time of radiotherapy treatment (RT) and the expected attained lifespan^22^:

5 $$LAR(D,e,a)={\int }_{a=e+L}^{75}EAR(D,e,a,s)\cdot \frac{S(a)}{S(e)}d{\rm{\alpha }}$$

where S(a) is the patient’s age at RT and S(e) is the attained age of survivors^23^. The Taiwanese population was used for the LAR calculation^24^.

### Statistical analysis

The statistical significance of differences between MLC-based plans and IRIS-based plans in terms of their DVH parameters was examined using the two-tailed Wilcoxon’s signed-rank test. Data analyses were performed using SPSS 22.0 (SPSS, Chicago, IL). In all analyses, *P* < 0.05 was taken to indicate statistical significance.

### Ethical approval and informed consent

All experimental protocols of this study were approved by the institutional review board (IRB) of Chang Gung Memorial Hospital. Due to this a retrospective study, the requirement for informed consent was waived in accordance with local laws and regulations (IRB approval No. 201701596B0 and 201802377B0). All of the studies and experiments were performed in accordance with specific regulations, rules, and guidelines.

## Results

Table 1 presents all of the treatment parameters and characteristics of the patients included in this study. The PTV coverage showed that IRIS- and MLC-based plans were equally effective (98.57 vs. 98.75, respectively), with higher values seen as a benefit. Equal coverage was seen in the ratio between MLC/IRIS ≅ 1.00. The statistical significance had different CI values (CI: 1.81 ± 0.26 for MLC and 1.92 ± 0.27 for IRIS, respectively, (*P* = 0.025), with a decrease in the value seen as a benefit.

**Figure 1:**
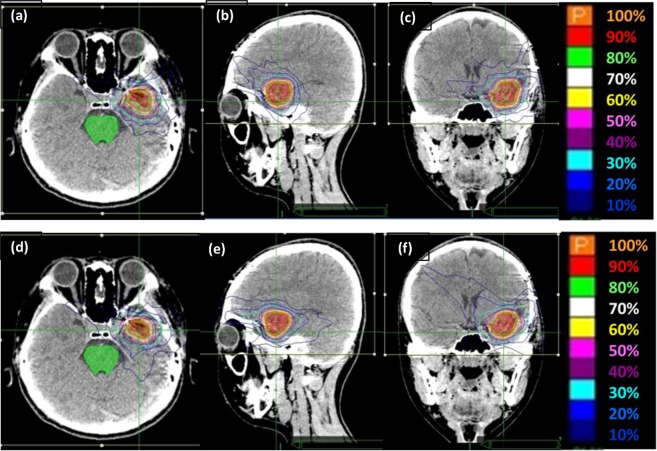
The isodose distributions on transverse, sagittal, and coronal views for one representative sample with (**a**–**c**) IRIS-based plans and (**d**–**f**) MLC-based plans. Abbreviations: MLC, Multileaf collimator; IRIS, Iris collimator.

Figure 1 shows the isodose distributions on coronal, transverse, and sagittal views within the IRIS-based and MLC-based plans for a single representative sample. Supplementary Table S2 shows the dose characteristics of both techniques. The mean doses were different for IRIS-based and MLC-based plans, e.g., soft tissues, 1.17 Gy vs. 1.06 Gy, respectively; brainstem, 1.15 Gy vs. 1.10 Gy, respectively. The ratio of MLC/IRIS plans indicated that the MLC had a slightly better sparing effect than IRIS for both soft tissue and the brainstem. Table 2 summarizes the dose falloff in both the IRIS-based and MLC-based techniques with the corresponding R_10%_ and R_50%_ values. Both techniques achieved a high dose gradient between the 50% prescription isodose line and the PTV periphery. In all cases, the IRIS technique had a mean R_50%_ value of 4.64, while the MLC technique had a mean R_50%_ value of 5.74. The MLC/IRIS ratio of R_50%_ did not exceed 1 (0.84, *P* < 0.05). This demonstrated a more effective dose falloff of the MLC-based plans compared to the IRIS-based plans. The IRIS and MLC techniques had mean R_10%_ values of 45.74 and 45.56, respectively; therefore, there was a decrease in distance to the 10% prescription isodose line provided that the MLC-based plan was used. Nevertheless, it should be noted that the difference was not statistically significant.

**Table 2:** Summary of dose falloff results in CyberKnife IRIS- and MLC-based plans.

	Number of Plans	R_50%_		R_10%_		Ratio(MLC/IRIS)		P-value (Wilcoxon’s signed-rank)	
		IRIS	MLC	IRIS	MLC	R_50%_	R_10%_	R_50%_	R_10%_
All cases	15	5.52 ± 1.41 (5.74)	4.64 ± 1.20 (4.56)	54.00 ± 25.60 (45.74)	54.99 ± 28.92 (45.56)	0.84	1.02	0.006	0.865
Target volume ≤1 cc	3	5.65 ± 1.17 (6.40)	5.95 ± 1.17 (5.86)	71.94 ± 18.97 (80.07)	95.02 ± 8.89 (98.13)	1.05	1.32	0.593	0.109
1 cc < Target volume ≤10 cc	7	5.40 ± 1.83 (4.95)	4.26 ± 1.18 (4.01)	57.85 ± 29.30 (48.38)	54.37 ± 24.94 (45.56)	0.79	0.94	0.028	0.612
Target volume >10 cc	5	5.61 ± 0.63 (5.74)	4.39 ± 0.49 (4.20)	37.84 ± 8.54 (37.15)	31.84 ± 9.62 (32.06)	0.78	0.84	0.043	0.043

Notes: Data represent the mean ± SD (median); Significance was defined as *P* < 0.05 on Wilcoxon’s Signed-Rank Test; R50%, represents the ratio of the volume covered by the 50% prescription isodose line of the maximum target dose (D50%) to the target volume; R10%, represents the ratio of the volume covered by the 10% prescription isodose line of the maximum target dose (D10%) to the target volume.

Abbreviations: MLC, Multileaf collimator; IRIS, Iris collimator; EAR, Excess Absolute Risk; CI, conformal index.

**Figure 2:**
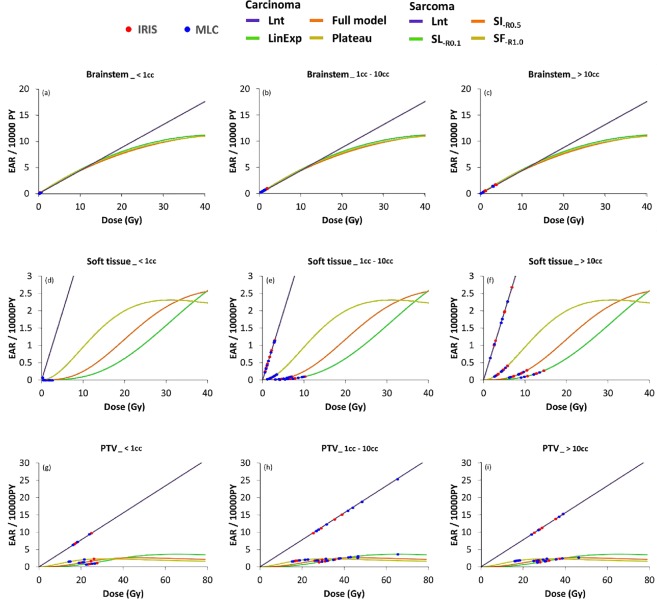
EAR (per 10,000 PY) as a function of RED for brainstem, soft tissue, and PTV using four different dose–response models in three different volume categories; dot plot for 15 patients stratified by the two techniques, i.e., MLC (blue dots) and IRIS (red dot), which presented the relationships between the corresponding DVH plots and the RED models. Notes: The EAR has units of excess cases per 10,000 person-years (PY)/Gy. Schneider dose–response model with repopulation/repair effects using Eq. 2 with a fixed limit of R; low repopulation (SL_-R0.1_) R = 0.1, intermediate repopulation (SI_-R0.5_) R = 0.5, full tissue recovery models (SF_-R1.0_) R = 1.0; The results showed that EAR for future secondary cancer was higher for patients who were younger at the time of radiation treatment. Significance (*P* < 0.05) was observed only in (**e**,**f**). Abbreviations: MLC, Multileaf collimator; IRIS, Iris collimator; Lnt, Linear-no-threshold dose–response model; LinExp, linear-exponential dose–response model; Plateau, Plateau dose–response model; Full, Schneider parameterization dose–response model.

EAR is presented in Fig. 2 as a RED function for soft tissue, brainstem, and PTV with the application of various models of dose–response type in three different volume categories; dot plots were used for the 15 patients after stratification using the IRIS techniques (red dots) and MLC techniques (blue dots). Therefore, it is possible to view the relationship between the RED models and the relevant DVH plot. EAR analyses indicated a significant difference in the soft tissue only for tumors with a volume exceeding 1 cc (1–10 cc and ≥10 cc. There were no significant differences between the two techniques for brainstem and PTV analyses regardless of the volume of the tumor.

**Figure 3:**
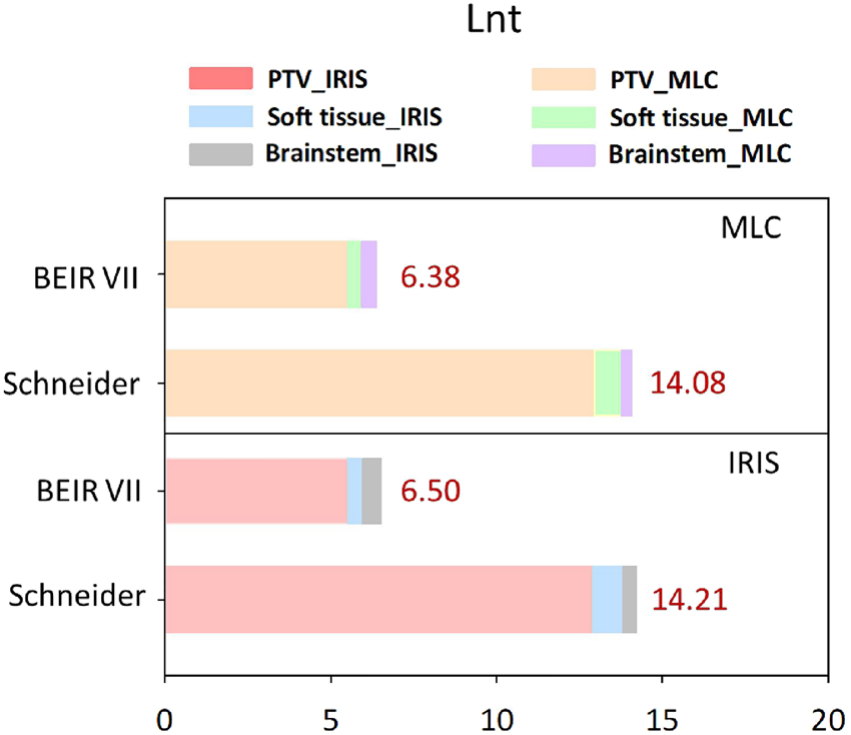
BEIR VII and Schneider Lnt Cumulative EAR of cancer for the three OARs at age 75 years stratified by the two techniques (MLC and IRIS). Note: Unit is per 10,000 person-years (PYs)/Gy. Abbreviations: MLC, Multileaf collimator; IRIS, Iris collimator; EAR, Excess absolute risk; Lnt, Linear no-threshold model; PTV, Planning target volume.

The overall values of EAR of Lnt cumulative type for the 15 patients are presented in Fig. 3 with the inclusion of Schneider’s models and BEIR VII to compare and contrast the use of MLC and IRIS plans. The results indicated a slight increase in SCR with use of the IRIS-based plan. The estimated difference in Lnt cumulative EAR using BEIR VII and Schneider’s model was approximately 220%.

**Figure 4:**
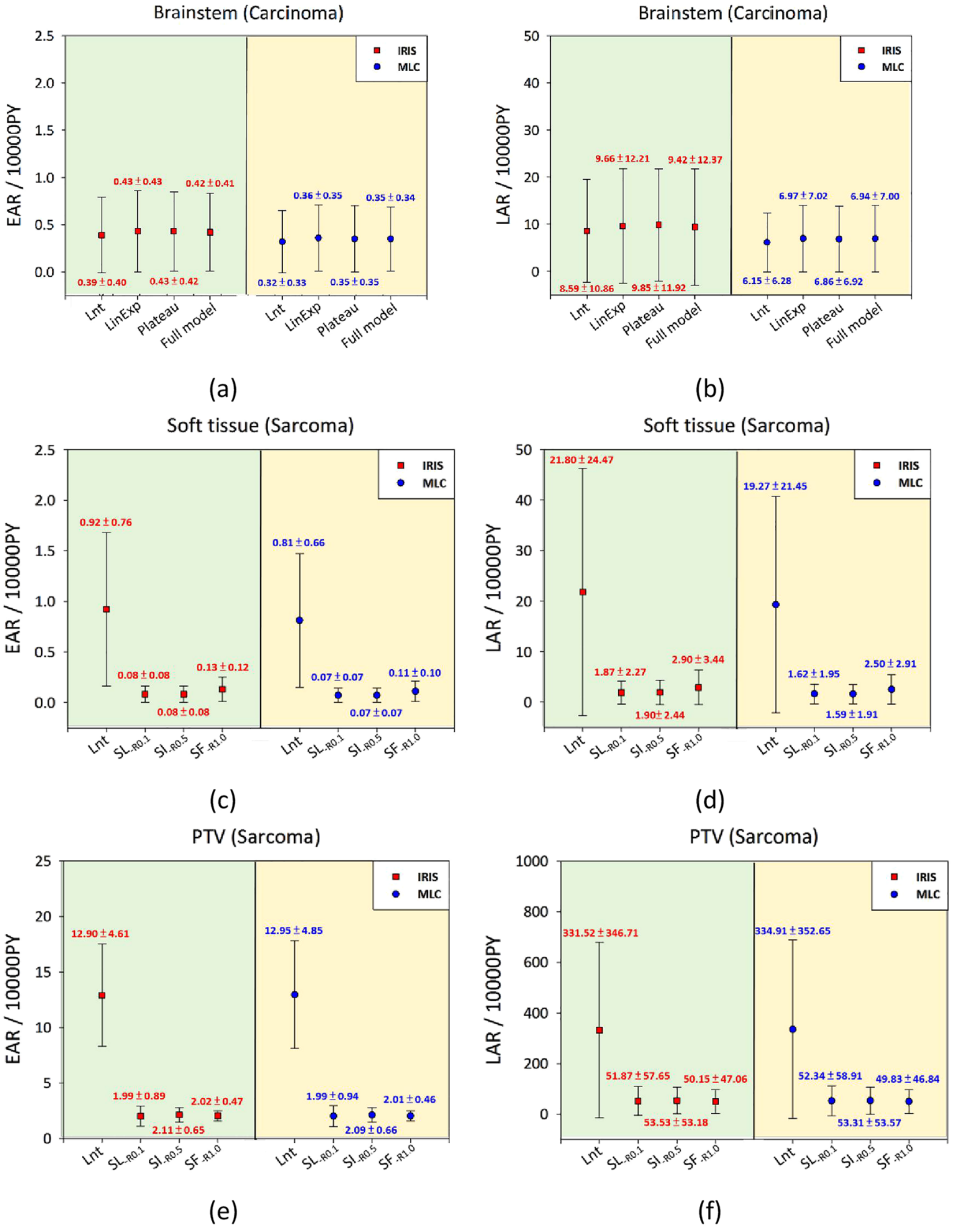
EARs and LARs of 15 patients (mean and standard deviation) for the brainstem and soft tissue, stratified by the two techniques (MLC and IRIS). Notes: (**a**) EAR, (**b**) LAR for brainstem, (**c**) EAR, (**d**) LAR for soft tissue, (**e**) EAR, (**f**) LAR for PTV. EAR has units of excess cases per 10,000 person-years (PY)/Gy. Lnt, Linear-no-threshold dose–response model; LinExp, linear-exponential dose–response model; Plateau, Plateau dose–response model; Full, Schneider parameterization dose–response model; Schneider dose–response model with repopulation/repair effects using Eq. 2 with a fixed limit of R; low repopulation (SL-_R0.1_) R = 0.1, intermediate repopulation (SI-_R0.5_) R = 0.5, full tissue recovery models (SF-_R1.0_) R = 1.0. Abbreviations: MLC, multileaf collimator; IRIS, Iris collimator; EAR, Excess absolute risk; LAR, lifetime attributable risk; OAR, organs-at-risk; Lnt, Linear-no-threshold model; PTV, Planning target volume.

The range of EAR/LAR is presented in Fig. 4a,b after the corresponding evaluation using the plateau, linear, full, and linear-exponential models for carcinoma induction in the brainstem. The range of EAR/LAR is presented in Fig. 4c,d after the corresponding calculation using the Schneider dose–response model for sarcoma induction in the soft tissue with use of RT techniques in the brain. The range of EAR/LAR is presented in Fig. 4e,f for the PTV. The average cumulative LAR values in the brainstem for patients at an attained age of 75 years were 6.73 vs. 9.38 per 10000 PYs/Gy for MLC and IRIS treatments, respectively. The values for soft tissue were 1.90 and 2.22 for MLC and IRIS, respectively. Both techniques had an average value of 51.8 for PTV. The LNT model was not included in this calculation for sarcoma analysis.

IRIS-based plans showed slightly higher values of mean EAR/LAR in comparison with MLC-based plans. However, this could not be applied to all patients. Supplementary Figs S1 and S2 show all of the details regarding the individual EAR and OED for each of the dose–response models. We concluded that EAR/LAR modality is dependent on the specific characteristics of each individual patient.

## Discussion

The results of the present study indicated that the CI values of the MLC-based plans were slightly lower than those of the IRIS-based plans. That is, the MLC-based plans showed a higher degree of conformity than the IRIS-based plans (*P* < 0.05). However, Jang *et al*. reported different results and showed that the CI values of the MLC-based plans were slightly higher than those of the IRIS-based plans. This discrepancy can be explained by the difference in number of targets, as the present study covered only one target. The MLC-based plans showed comparable PTV coverage to the IRIS-based plans (MLC/IRIS ratio ≅ 1) with a decrease in time for delivery. The MLC technique has a number of advantages, but the most significant characteristic feature making it superior to the IRIS-based plans is the decrease in time of delivery^3^.

For beam collimation in our IRIS-based plan, treatment plans were generated by combined use of a small cone and a large cone (two-cone plan) instead of a small single cone. This reduced the MU and delivery time for treatment^25^. However, this application may cause reduced dose falloff and conformal dose distribution compared to the MLC-based plan (Table 2). In such cases, the R_50%_ value ratio for MLC-based plans and IRIS-based plans was 0.84. This was a demonstration of the more efficient gradient of the high dose obtained for the MLC-based plans between the 50% prescription isodose line and the target periphery. With the exception of the small (1 cc) targets, the R_50%_ ratio between the MLC-based plans and IRIS-based plans exceeded 1. This was an indication that the small targets for IRIS-based plans had a slightly better R_50%_ in comparison with the MLC-based plans. Therefore, the IRIS-based plans showed better suitability for small lesions due to the collimators’ circle shape.

The MLC/IRIS ratios of mean and maximum doses were 0.88 and 0.85 in the brainstem, respectively, 0.90 and 1.01 in soft tissue, respectively, and 1.00 and 1.01 in PTV, respectively. If the ratio does not exceed 1, it indicates that the dose of the MLC-based plans to OAR is lower than that of IRIS-based plans. It should be noted that the ratio did not show any statistically significant difference between the two methods.

The present study analyzed all possible scenarios of SCR in patients exposed to CK SRS, as there is still lack of valid information and knowledge regarding the dose–response models. This study applied the plateau, linear, full, and linear-exponential models for carcinoma analysis. The results of irradiated contribution after applying the four different models showed almost identical OED as the dose used was small and all of the models were in the linear low-dose range. The study revealed some differences in OED between the relationships of linear-exponential and linear dose–response types that did not exceed 5%. The difference between the plateau and linear models did not exceed 18%, while the difference between the full and linear models was less than 7%. Schneider *et al*. reported different results, indicating that the actual dose–response curve for the induced cancer was expected to be between the linear and linear-exponential models^26^.

The BEIR VII model did not consider the distribution of non-uniform dose, high-dose radiation-induced cancer, or biologically effective dose (BED) correction. The epidemiological statistics of the atomic bomb explosion are based on one-shot low-dose radiation. Therefore, the cancer induction model is also a linear dose function, and the BEIR VII model can be expected to underestimate the SCR. Schneider’s model is closer to the population discussed in this study than the atomic bomb explosion data. However, the data fit is based on fractional radiotherapy. For the hypofraction RT explored in this study, it is necessary to use BED to convert to the same radiation biological effect to evaluate reasonably the incidence of SRS-induced SCR. In one patient as an example, the PTV mean dose before conversion was 20 Gy, while the mean dose was 56 Gy after BED conversion. The radiation biological effect was the same for 20 Gy in SRS and 56 Gy in fractional radiotherapy. Figure 3 shows that if high-dose RT is evaluated directly based on the atomic bomb explosion data, it will not be able to deal with cell radiation biological effects and inhomogeneity of dose distribution on SCR. Based on these considerations, the Lnt cumulative EAR difference between BEIR VII and Schneider’s model is about 220%.

The present study indicated insignificant growth of the Lnt cumulative EAR (BEIR VII) for IRIS-based models and a 17% increase in the Schneider Lnt model (see Fig. 3). The calculation did not include the PTV. According to the average estimations, the values of the IRIS-based plans in the case of carcinoma induction had EAR that was 20% higher and LAR that was 30% higher than those of MLC-based plans in all of the four SCR models. Schneider’s SCR models for soft tissue sarcoma induction had about 15% higher general EAR/LAR values (∆% = (IRIS− MLC)/MLC × 100%). The results of carcinoma analysis for the brainstem demonstrated compatible values in linear-exponential, linear, full parameterization, and plateau dose–response models. For sarcoma analysis with regard to PTV and soft tissue, the results of EAR/LAR using three models also showed compatible values in low repopulation, intermediate repopulation, and full tissue recovery model. The only exception was the linear-no-threshold (Lnt) model that indicated EAR/LAR overestimation. It was assumed that the Lnt model is not suitable for sarcoma analysis of PTV and soft tissue. Therefore, it is not possible to choose one suitable method as a gold standard for analysis of SCR associated with RT. Therefore, SCR study requires references obtained by comparison between the models.

The SCR in epidemiological studies is affected by a number of different factors, in particular, the age at initial exposure and RT dose^27^. The present study population included those who had been initially exposed at different ages (range, 15–64 years), with Patient 1 as the youngest participant and Patient 13, with identical OED values. The analysis demonstrated a significant (sixfold) difference between these patients. Younger patients had SCR as a late complication, and long-term survivors required careful attention after treatment for benign intracranial tumors. Paganetti *et al*. reported similar results, indicating a decrease in average age of RT patients, with the introduction of new complex techniques for treatment bringing various issues related to secondary cancers induced by radiation^28^.

This study had a number of limitations. First, the dose calculations were based on commercially available treatment planning systems, which could therefore produce some inaccuracies in the estimates related to their own calculation algorithms^17–20^. Second, the study population was small. To improve the performance of the SCR models and decrease the uncertainties, longer term epidemiological studies in larger populations are required^2,16^. However, there have been no previous clinical analyses of secondary cancer induction after CK treatment due to time limitations and restricted clinical availability, as such studies require decades rather than years to complete.

## Conclusion

MLC-based plans showed slightly greater conformity, SCR reduction, and dose gradient than IRIS-based plans. However, the increase in target volume resulted in an increase in the probability of SCR. The average estimates showed an approximately 25% higher overall EAR/LAR value in the framework of the IRIS-based plans for carcinoma in four SCR models in comparison with MLC-based plans. In addition, they showed approximately 15% higher values in four Schneider SCR models for sarcoma. The small OED in the model of the low-dose range was linear. EAR analyses did not show any statistically significant differences between the techniques for the brainstem and PTV analyses regardless of tumor volume. EAR showed statistically significant differences in the soft tissue according to tumor volume (1–10 cc and ≥10 cc). However, there is no gold standard for SCR analysis, and selection of the model for effective estimation should take into consideration all of the dose–response factors.

## Supplementary information

Supplementary

Dataset_1

Dataset_2

## Data Availability

The data that support the findings of this study are available within the article and its Supplementary Materials.
