# Supplementary material for: Plan Quality and Secondary Cancer Risk Assessment in Patients with Benign Intracranial Lesions after Radiosurgery using the CyberKnife M6 Robotic Radiosurgery System
Source: Sci Rep. 2019 Jul 9;9:9953. doi: 10.1038/s41598-019-46133-8 (PMC6616465; doi:10.1038/s41598-019-46133-8)
Supplement: Supplementary file 1 — Supplementary [file 41598_2019_46133_MOESM1_ESM.docx]

**Plan Quality and Secondary Cancer Risk Assessment in Patients with Benign Intracranial Lesions after Radiosurgery using the CyberKnife M6 Robotic Radiosurgery System**

Jen-Hong Lan, MS^1,2,†^, Chin-Shiuh Shieh, PhD^1^, Chao-Hong Liu, I-Chun Cho, PhD^1,2,†^, MD^1,3^, I-Hsing Tsai, MS^1^, Long-Chun Chen, MS^1^, Pei-Ju Chao, PhD^1,2^, Hsiao-Fei Lee, PhD^1,2^, Yu-Jie Huang, MD PhD^2,*^, Tsair-Fwu Lee, PhD^1,2,4,*^,

^1^ Medical Physics and Informatics Laboratory of Electronics Engineering, National Kaohsiung University of Science and Technology, Kaohsiung, Taiwan, ROC

^2^ Department of Radiation Oncology, Kaohsiung Chang Gung Memorial Hospital and Chang Gung University College of Medicine, Kaohsiung, Taiwan, ROC

^3^ Department of Dermatology, Kaohsiung Yuan’s General Hospital, Kaohsiung, Taiwan, ROC.

^4^ Graduate Institute of Clinical Medicine, Kaohsiung Medical University, Kaohsiung, Taiwan, ROC

| **Supplementary** |
| --- |

| **Supplementary Table S1**. Summary of risk parameters used for the corresponding dose-response models | | | | | | | | | | | | |
| --- | --- | --- | --- | --- | --- | --- | --- | --- | --- | --- | --- | --- |
| Carcinoma | | | | | | | | | | | | |
| Organ | Lnt | LinExp | | | Plateau | | | Full model | | | Age parameters | |
|  | β | β | α | R | β | α | R | β | α | R | γ_e_ | γ_a_ |
| Brain stem | 0.44 | 0.51 | 0.009 | 0 | 0.51 | 0.021 | 1 | 0.51 | 0.018 | 0.93 | -0.024 | 2.38 |
| Sarcoma | | | | | | | | | | | | |
| Organ | Lnt | Low repopulation | | | Intermediate repopulation | | | Full tissue recovery | | | Age parameters | |
|  | β | β | α | R | β | α | R | β | α | R | γ_e_ | γ_a_ |
| Soft tissue | 0.39 | 3.3 | 0.040 | 0.1 | 0.6 | 0.060 | 0.5 | 0.35 | 0.093 | 1 | -0.013 | -0.56 |
| **Notes:** β is used for EAR calculation only; β within the α/β ratio is calculated from a based on α/β =3 Gy for all tissues; R, cell repopulation parameter; α, cell killed parameter; Plateau, Plateau dose response model; Full model, Schneider’s parameterization (full) model; γe and γa, modifying factors for age;  **Abbreviations:** Lnt, Linear-no-threshold dose response model; LinExp, linear-exponential dose response model; | | | | | | | | | | | | |

| Supplementary Table S2. The dose characteristics | | | | | | | | | | |
| --- | --- | --- | --- | --- | --- | --- | --- | --- | --- | --- |
| modality | Mean Dose (Gy) | | | | | Max Dose (Gy) | | | | |
|  | IRIS | MLC | Ratio  (MLC/IRIS) | ∆ (%) | p | IRIS | MLC | Ratio  (MLC/IRIS) | ∆ (%) | p |
| Optic Chiasm | 1.41 | 0.91 | 0.65 | 54.24 | 0.047 | 2.76 | 2.09 | 0.76 | 32.47 | 0.028 |
| White Matter | 0.99 | 0.88 | 0.89 | 12.63 | 0.139 | 14.12 | 14.20 | 1.01 | -0.54 | 0.767 |
| Right Eye | 0.04 | 0.04 | 1.00 | 0.28 | 0.721 | 0.38 | 0.20 | 0.53 | 95.72 | 0.093 |
| Right Optic Nerve | 0.53 | 0.41 | 0.77 | 29.92 | 0.139 | 2.13 | 1.53 | 0.72 | 39.52 | 0.013 |
| Right Lens | 0.03 | 0.03 | 1.00 | -13.70 | 0.333 | 0.03 | 0.04 | 1.33 | -10.54 | 0.575 |
| Left Eye | 0.04 | 0.07 | 1.75 | -39.65 | 0.169 | 0.33 | 0.31 | 0.94 | 4.27 | 0.799 |
| Left Optic Nerve | 0.63 | 0.70 | 1.11 | -10.35 | 0.878 | 2.29 | 1.87 | 0.82 | 22.03 | 0.169 |
| Left Lens | 0.03 | 0.05 | 1.67 | -43.12 | 0.241 | 0.03 | 0.06 | 2.00 | -45.91 | 0.203 |
| Cerebellum | 1.93 | 1.86 | 0.96 | 3.91 | 0.169 | 11.74 | 11.76 | 1.00 | -0.14 | 0.721 |
| Brain Stem | 1.18 | 1.04 | 0.88 | 13.52 | 0.281 | 5.08 | 4.31 | 0.85 | 18.05 | 0.002 |
| Pituitary Gland | 2.05 | 1.82 | 0.89 | 12.39 | 0.386 | 3.33 | 3.07 | 0.92 | 8.32 | 0.508 |
| Soft tissue | 1.46 | 1.31 | 0.90 | 10.87 | 0.009 | 18.31 | 18.51 | 1.01 | -1.11 | 0.410 |
| PTV | 17.32 | 17.32 | 1.00 | 0.03 | 0.609 | 18.75 | 18.91 | 1.01 | -0.79 | 0.866 |
| Notes: ∆ = (IRIS- MLC)/ MLC* 100 %; Positive, IRIS > MLC; Negative, MLC > IRIS; With a p-value of 0.05 from the Wilcoxon Signed Ranked Test;  Abbreviations: MLC, multi-leaf collimator; IRIS, Iris collimator; EAR, Excess absolute risk; LAR, lifetime attributable risk; OAR, organs-at-risk; Lnt, Linear-no-threshold model; PTV, Planning target volume. | | | | | | | | | | |

| 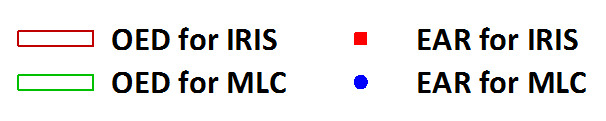  **Carcinoma Sarcoma Sarcoma**  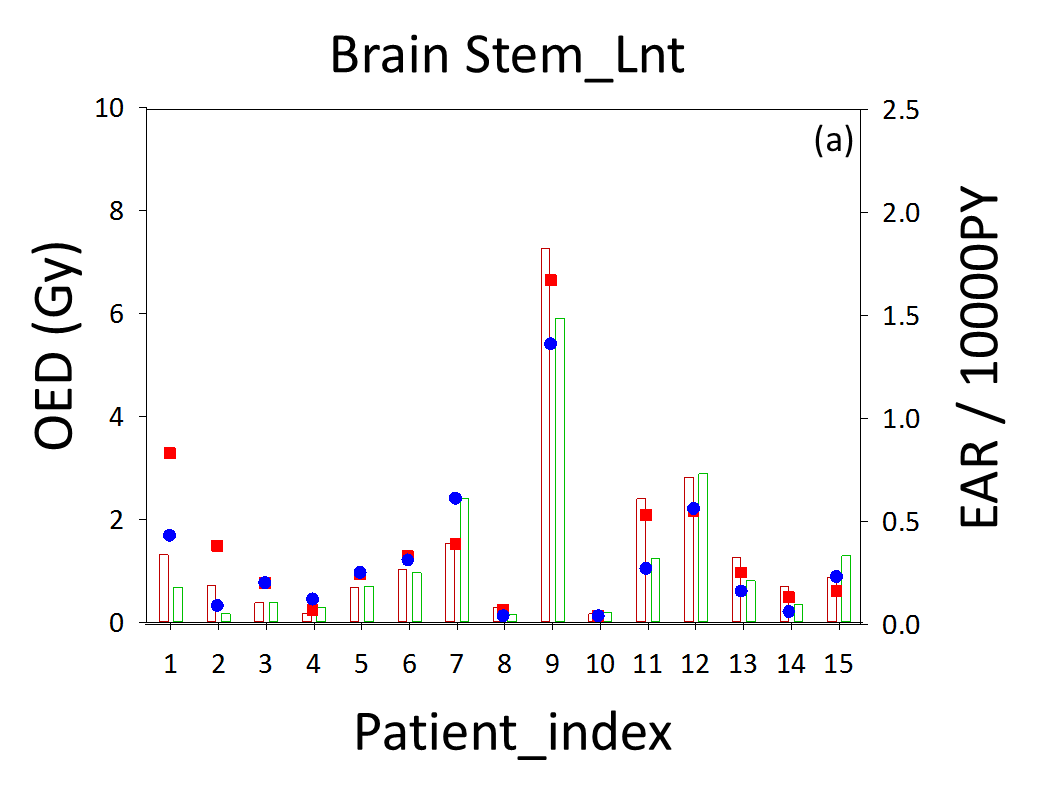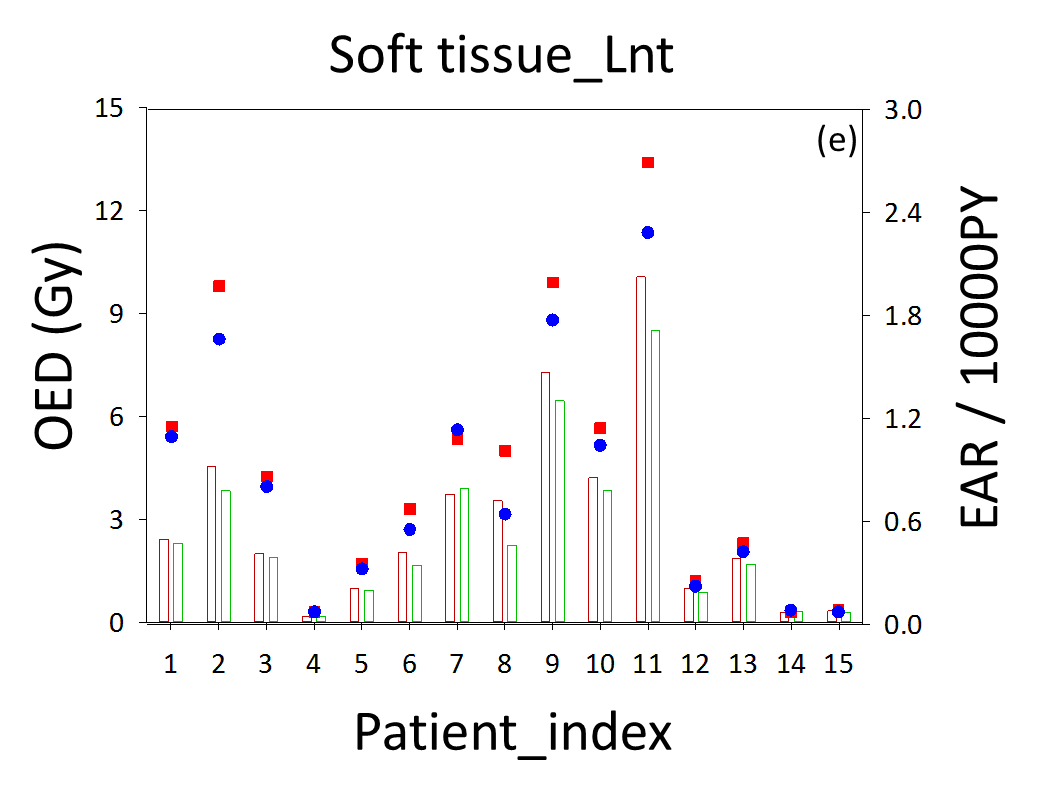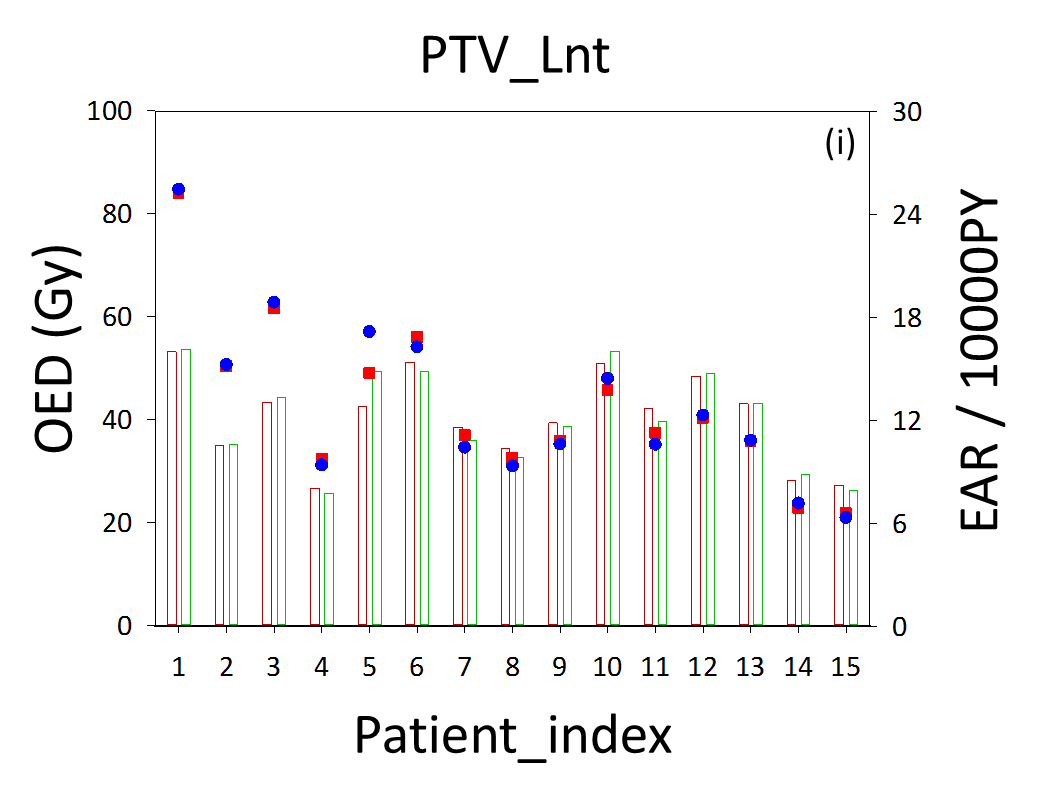  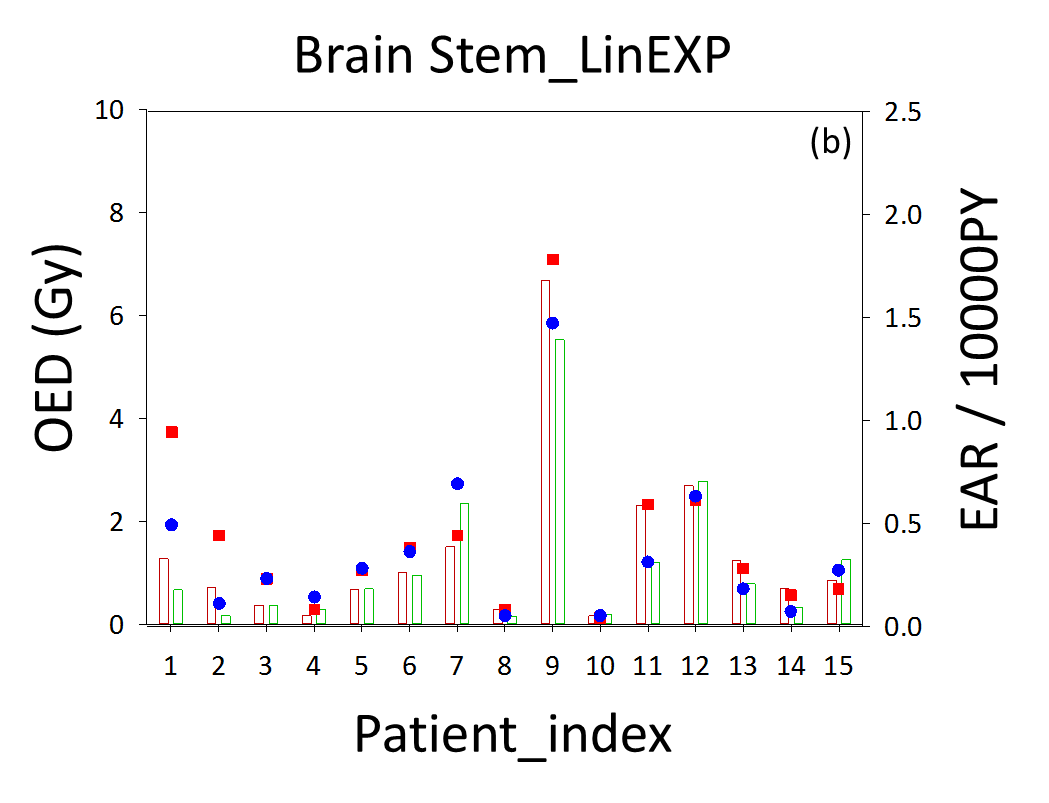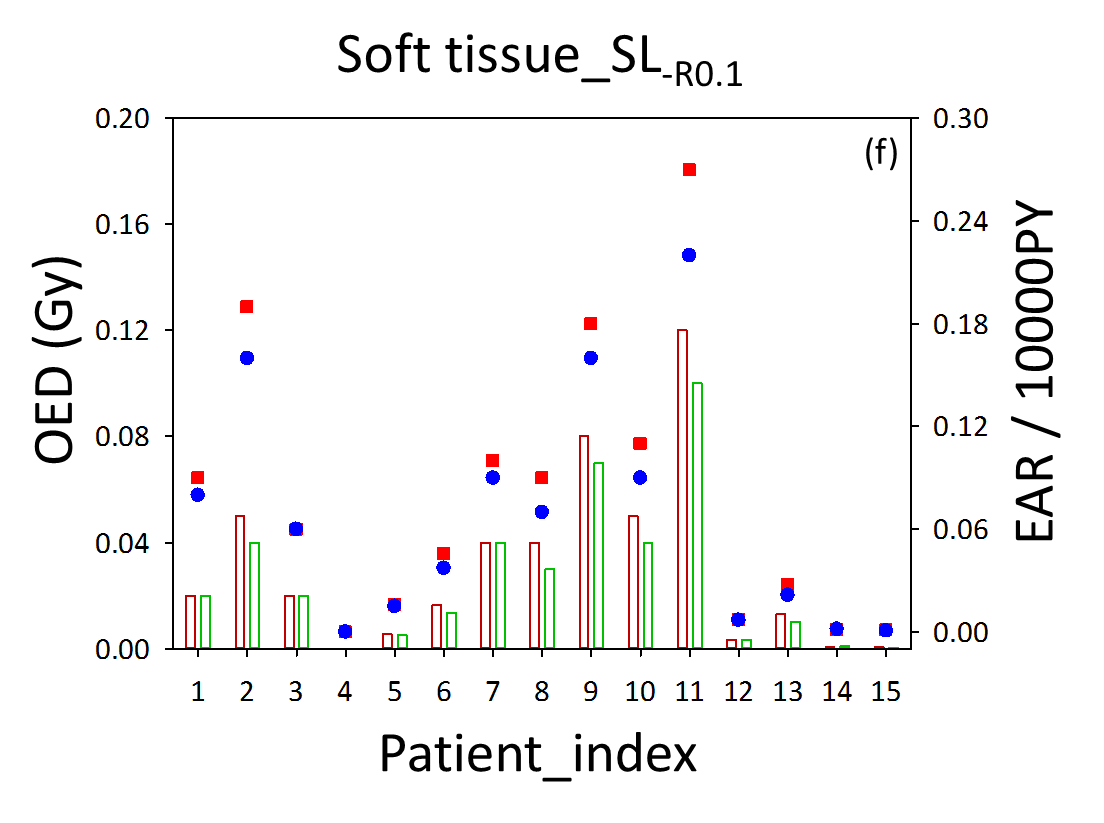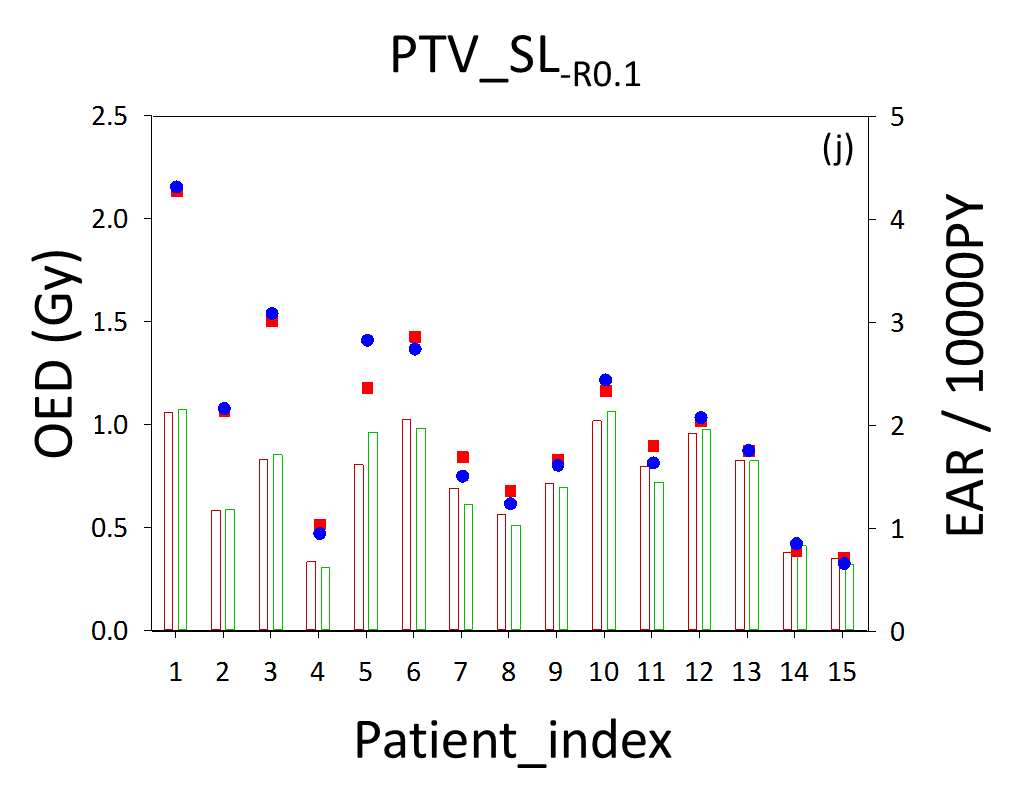  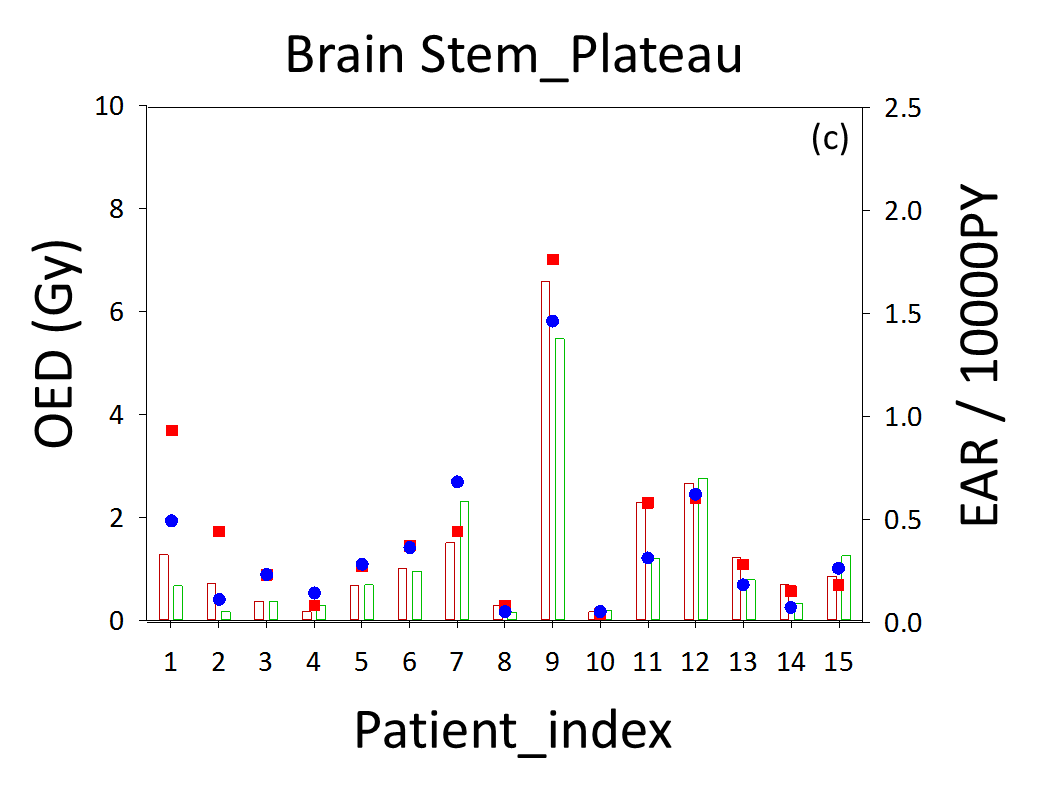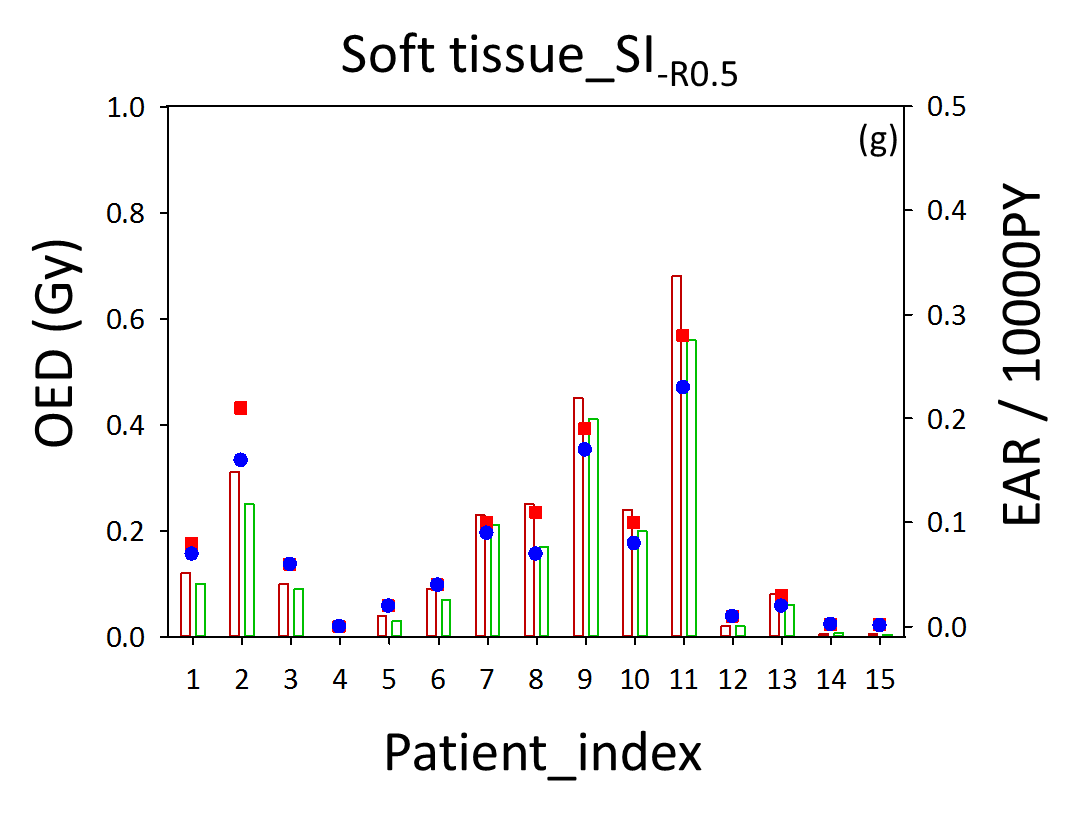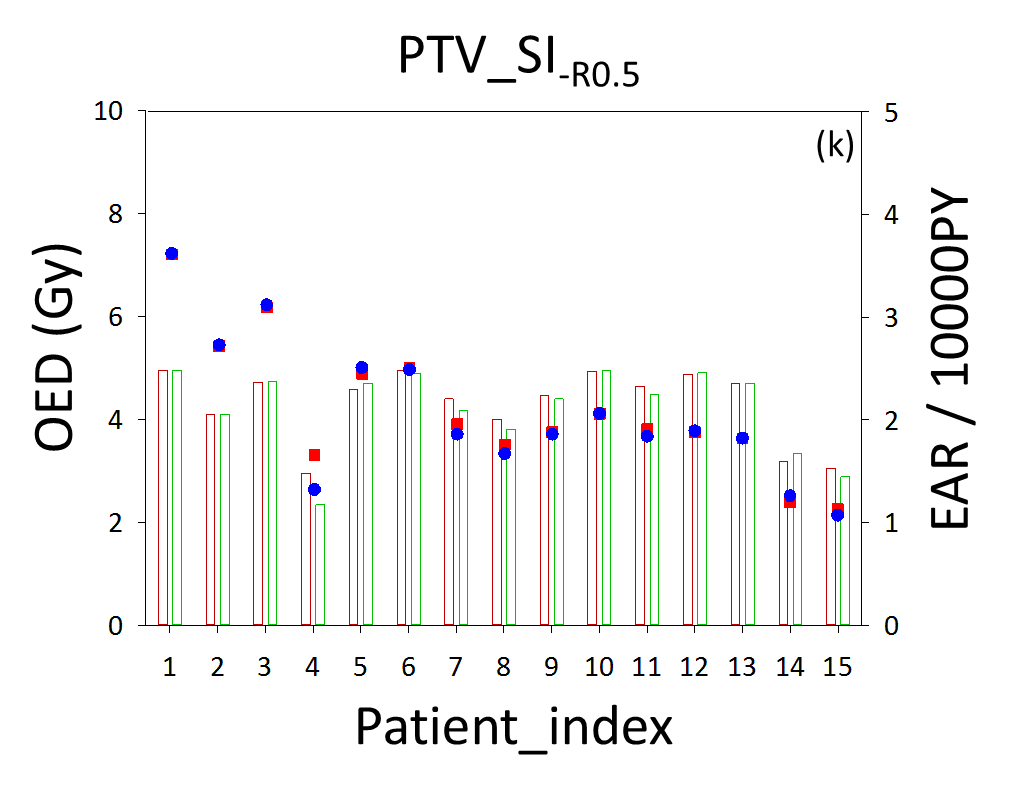  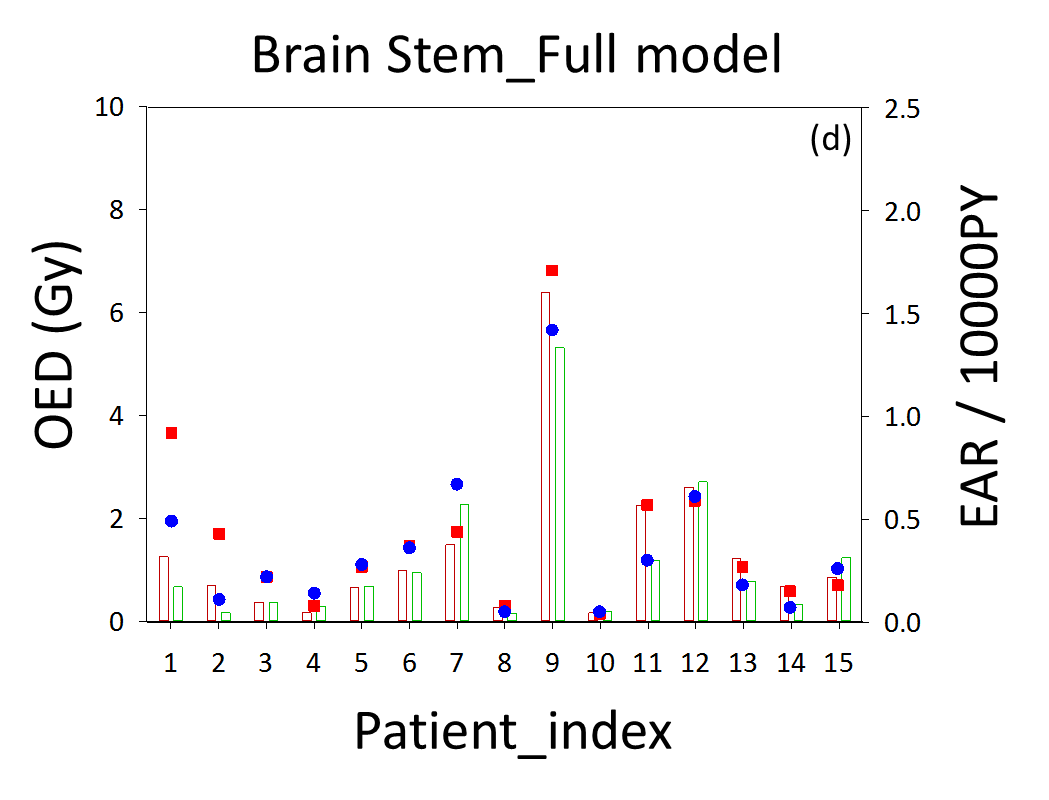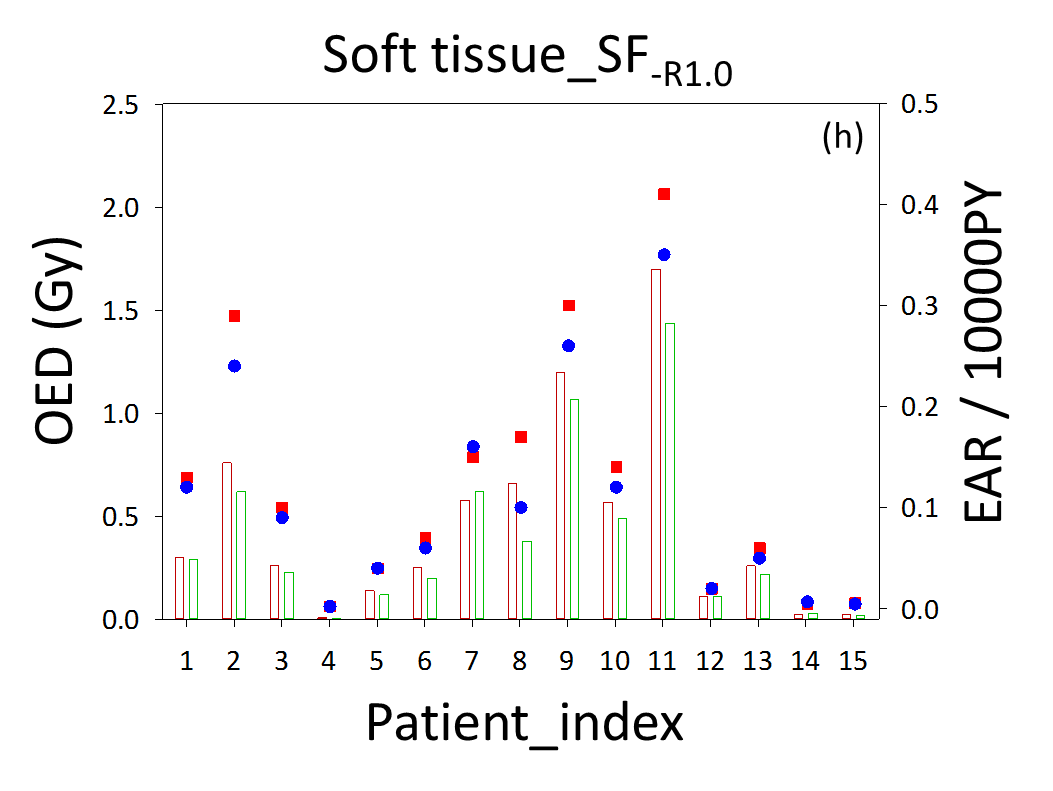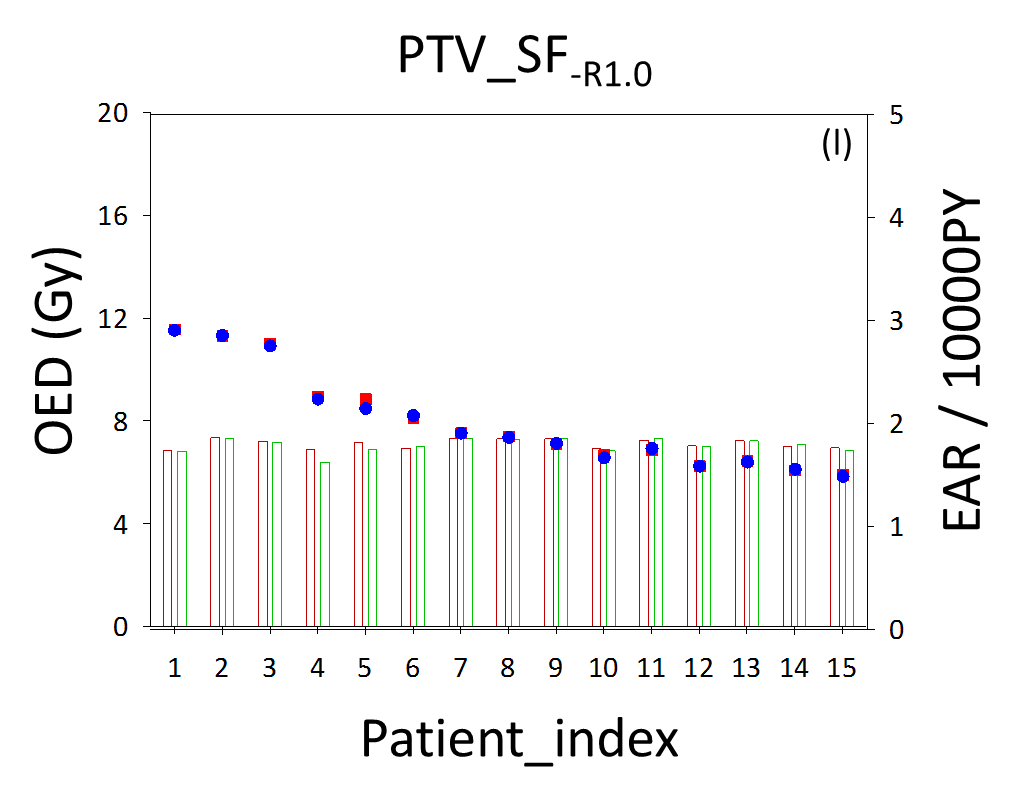  **Supplementary Figure S1**. OED/EAR of 15 patients using corresponding dose-response model stratified by the two techniques of MLC and IRIS. (a-d) for brain stem; (e-h) for soft tissue; (i-l) for PTV.  **Notes:** The EAR has units of excess cases per 10,000 person-years (PY)/Gy. Schneider dose-response model with repopulation/repair effects by using Eq.2 with a fixed limit of R; low repopulation (SL_-R0.1_) R = 0.1, intermediate repopulation (SI_-R0.5_) R = 0.5, full tissue recovery models (SF_-R1.0_) R = 1.0; The results show that the younger a patient is at the time of radiation treatment, the higher the EAR is for a future secondary cancer. Patients were ordered by age from young to old.  **Abbreviations:** MLC, multi-leaf collimator; IRIS, Iris collimator; Lnt, Linear-no-threshold dose response model; LinExp, linear-exponential dose response model; Plateau, Plateau dose response model; Full, Schneider parameterization dose-response model; PTV, Planning target volume. |
| --- |
| 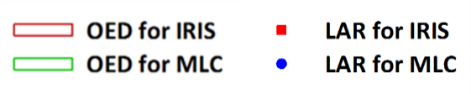 |
| **Carcinoma Sarcoma Sarcoma** |


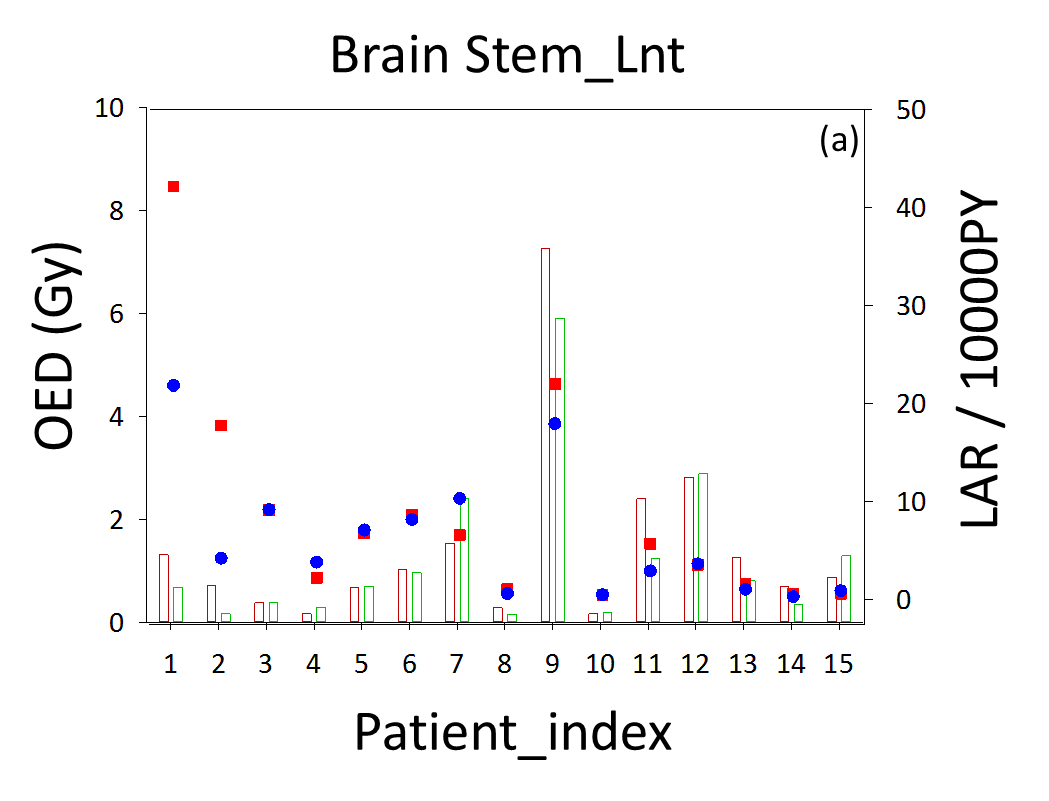

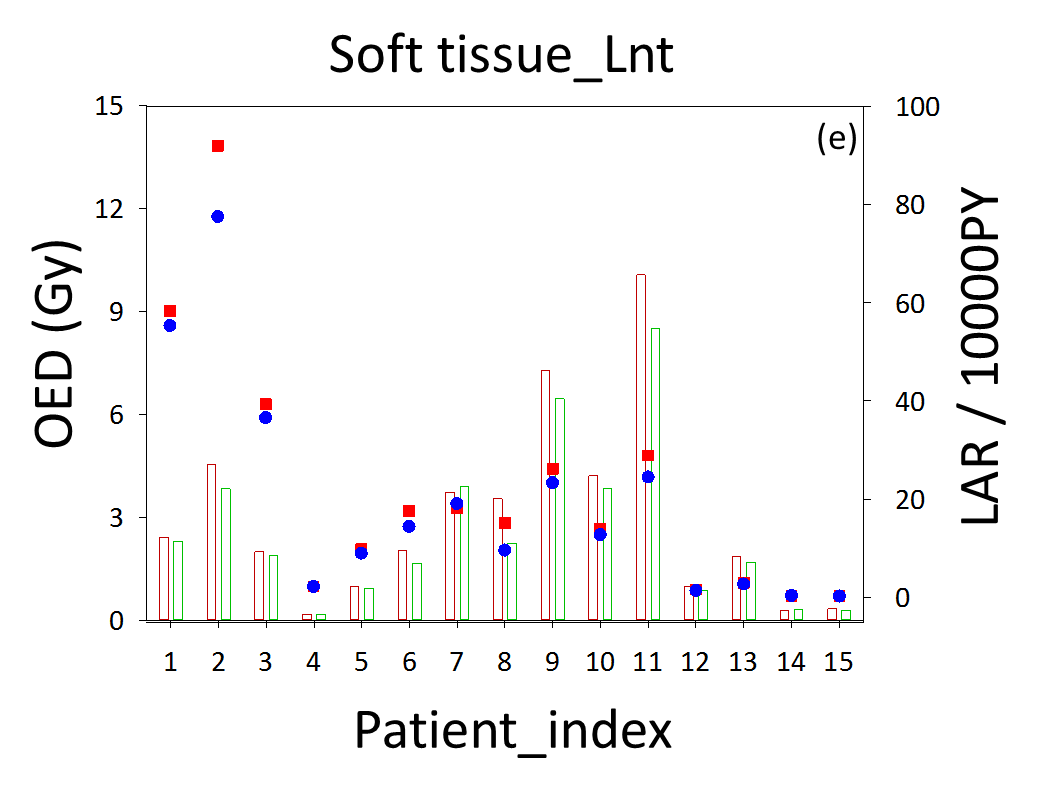

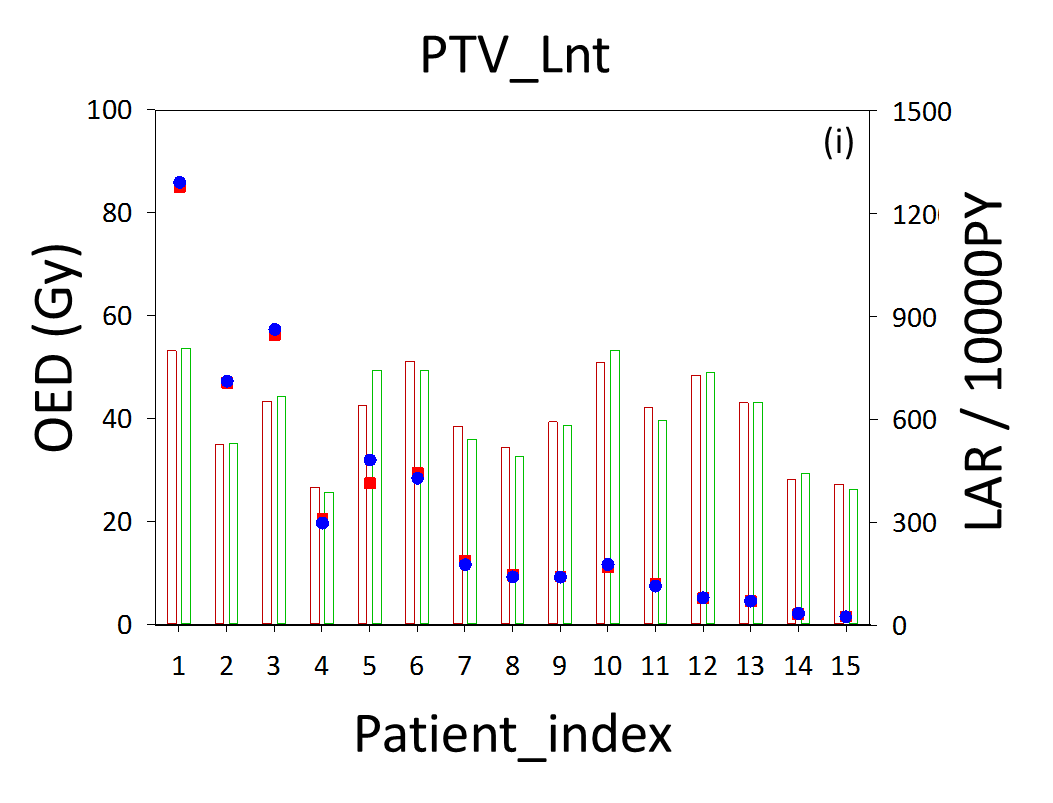


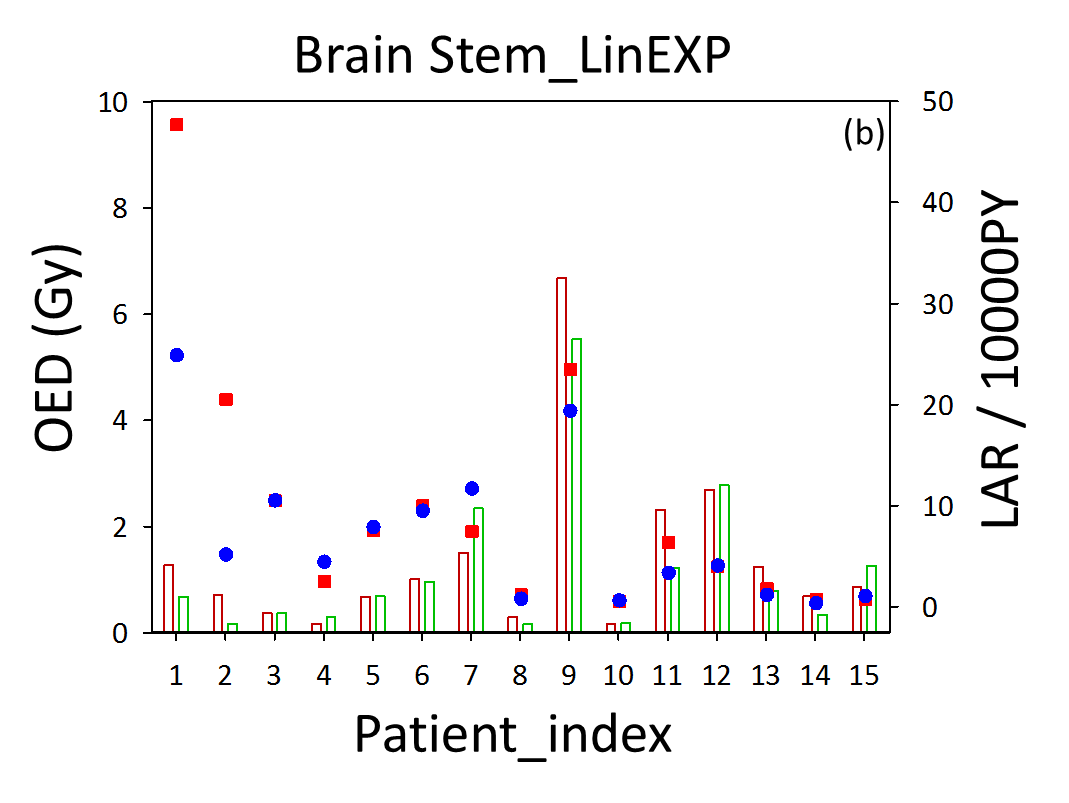

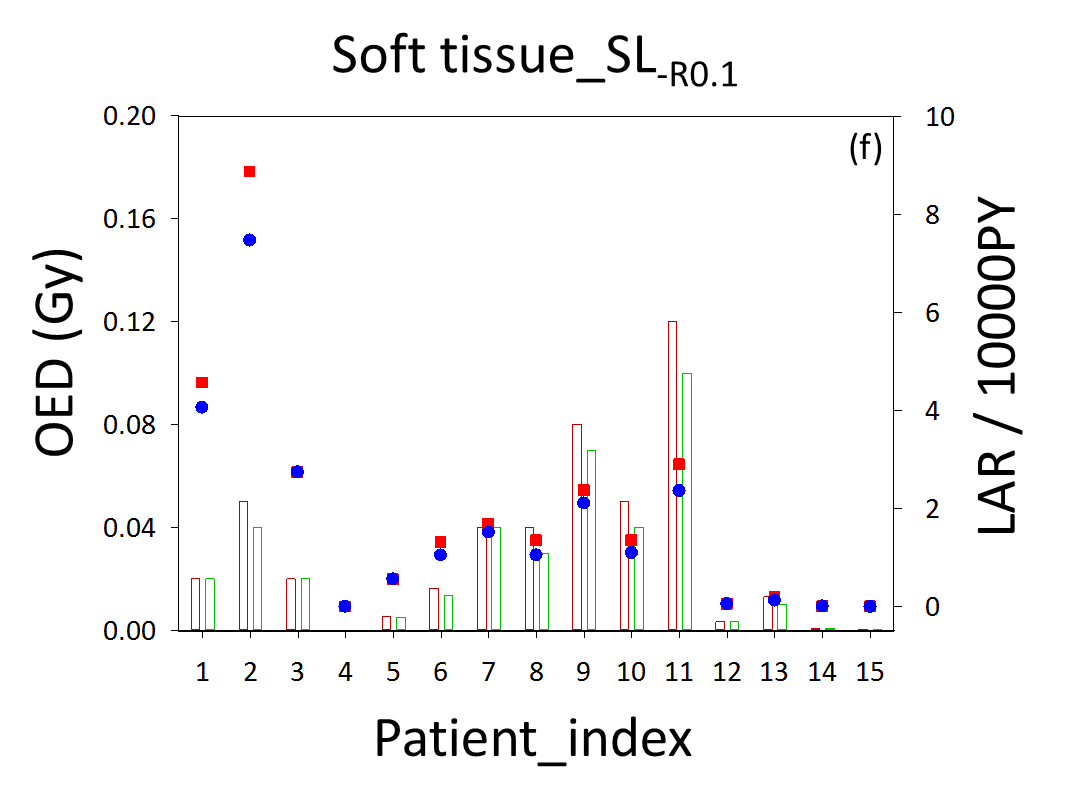

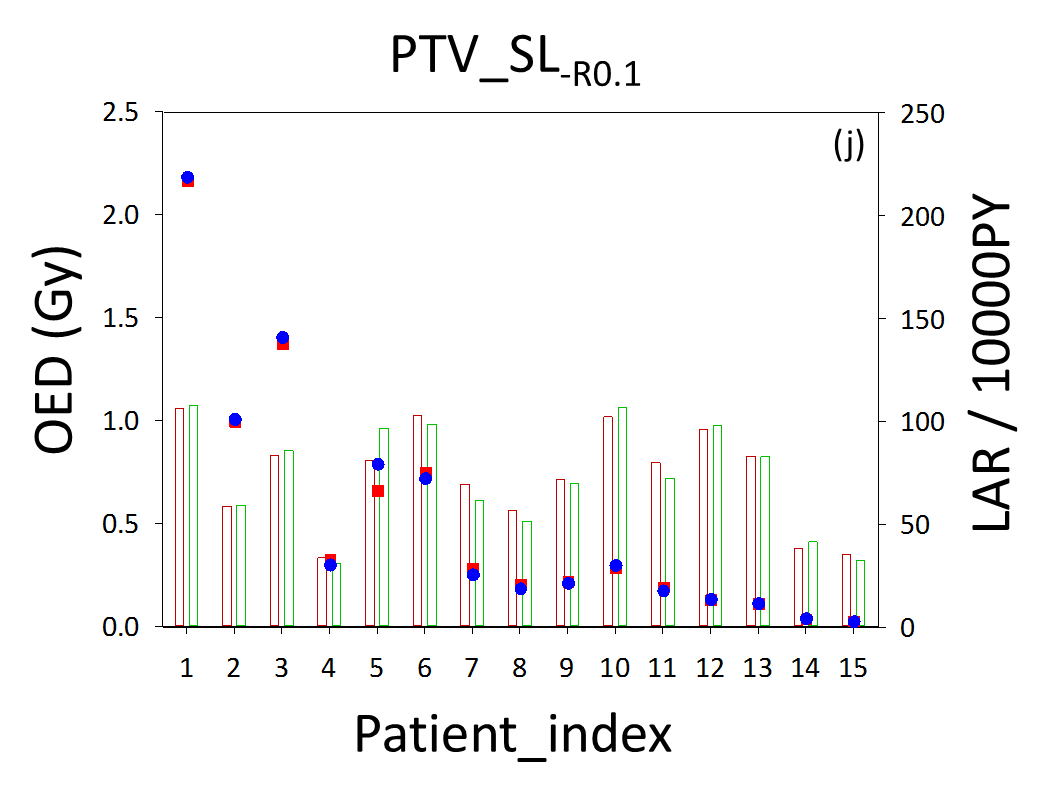


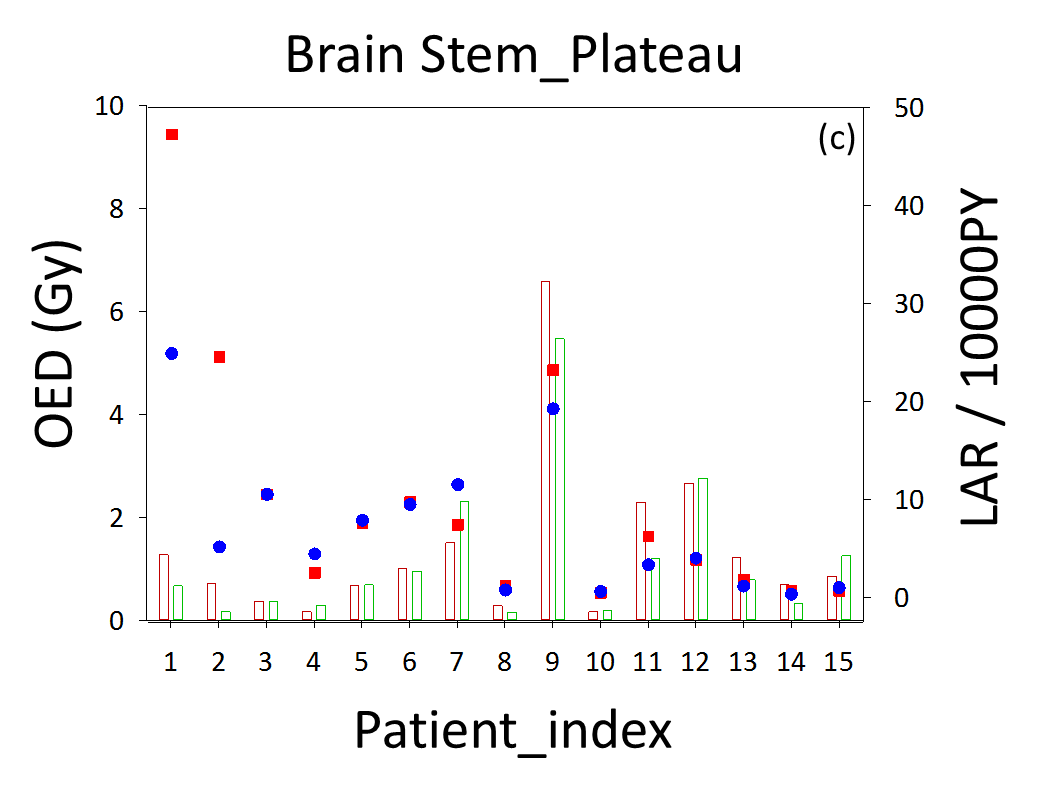

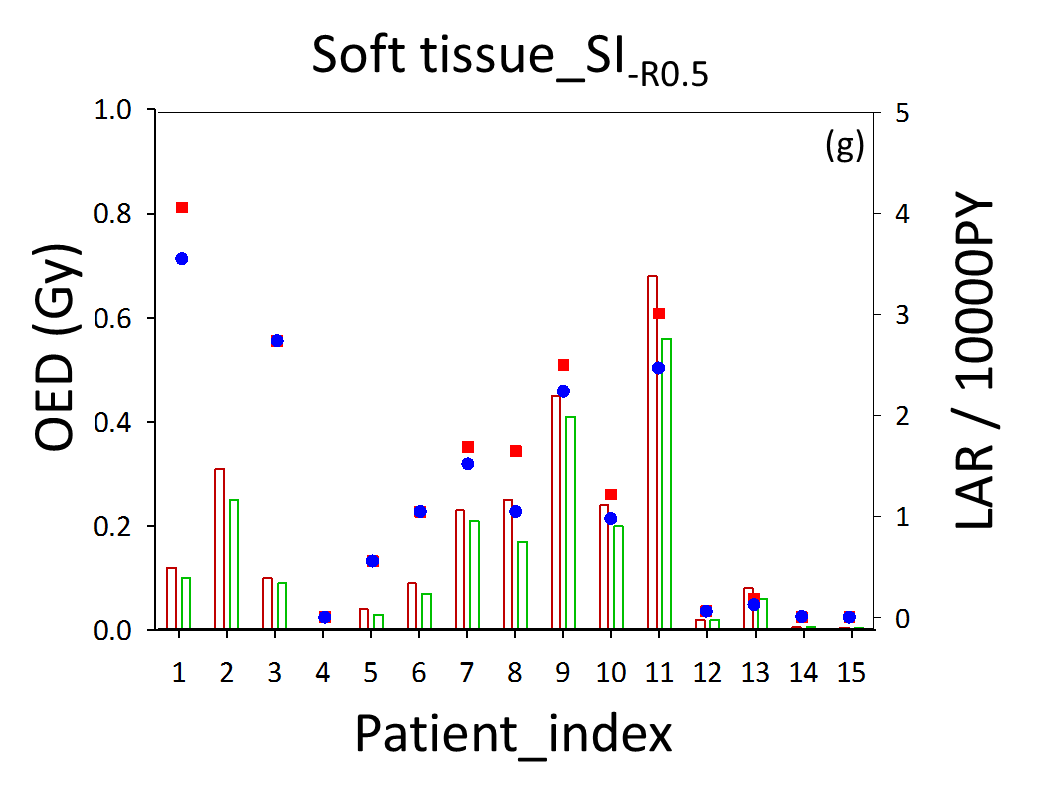

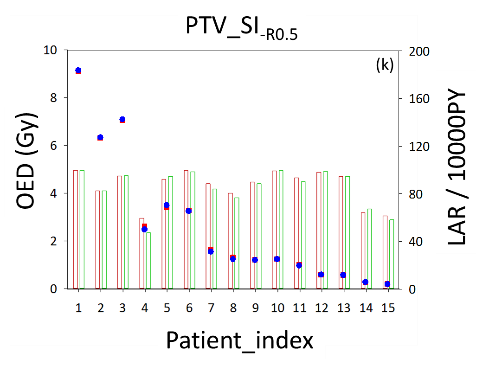


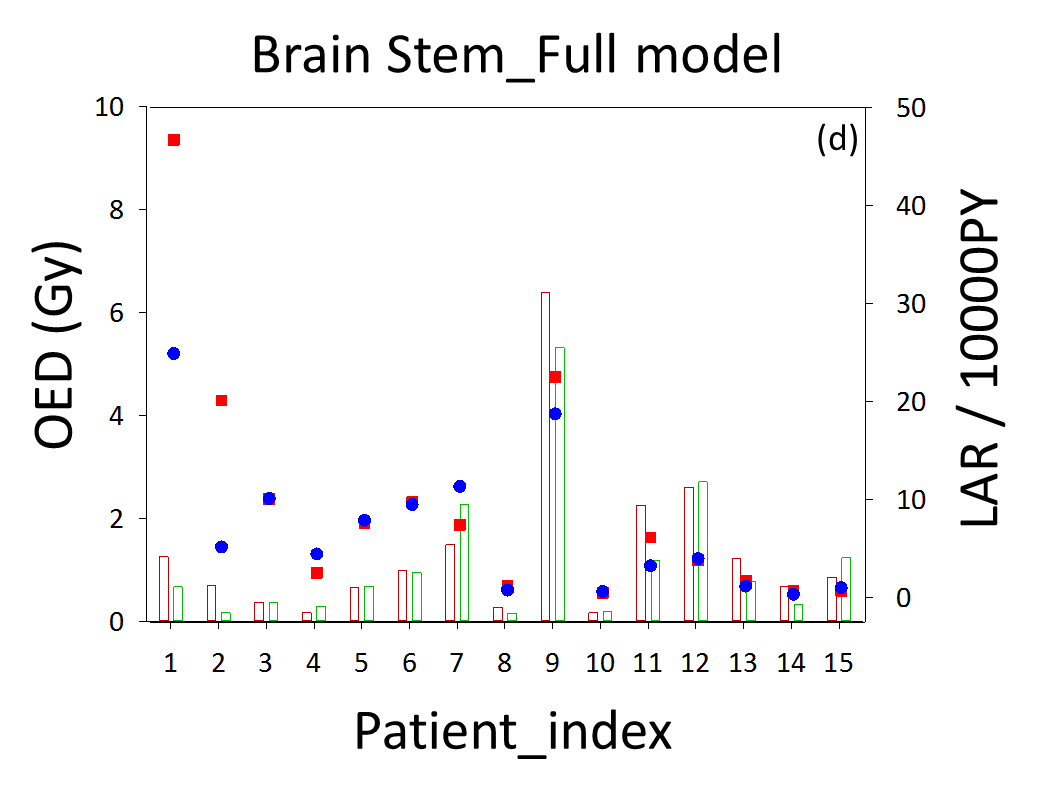

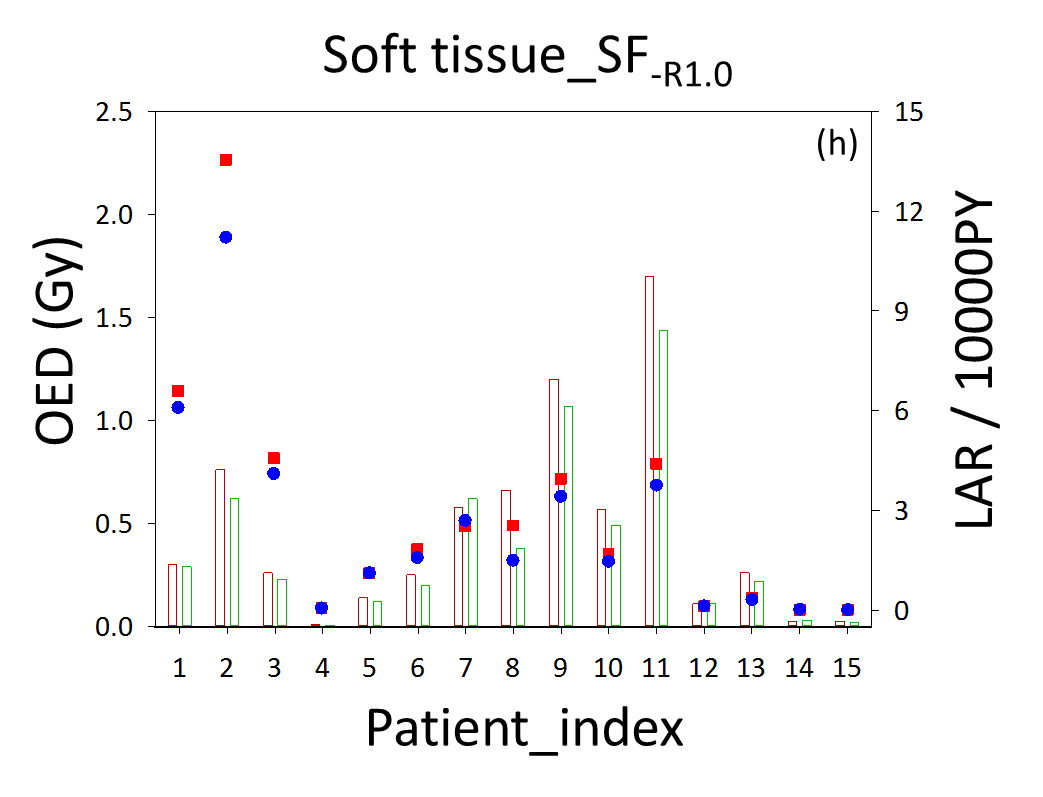

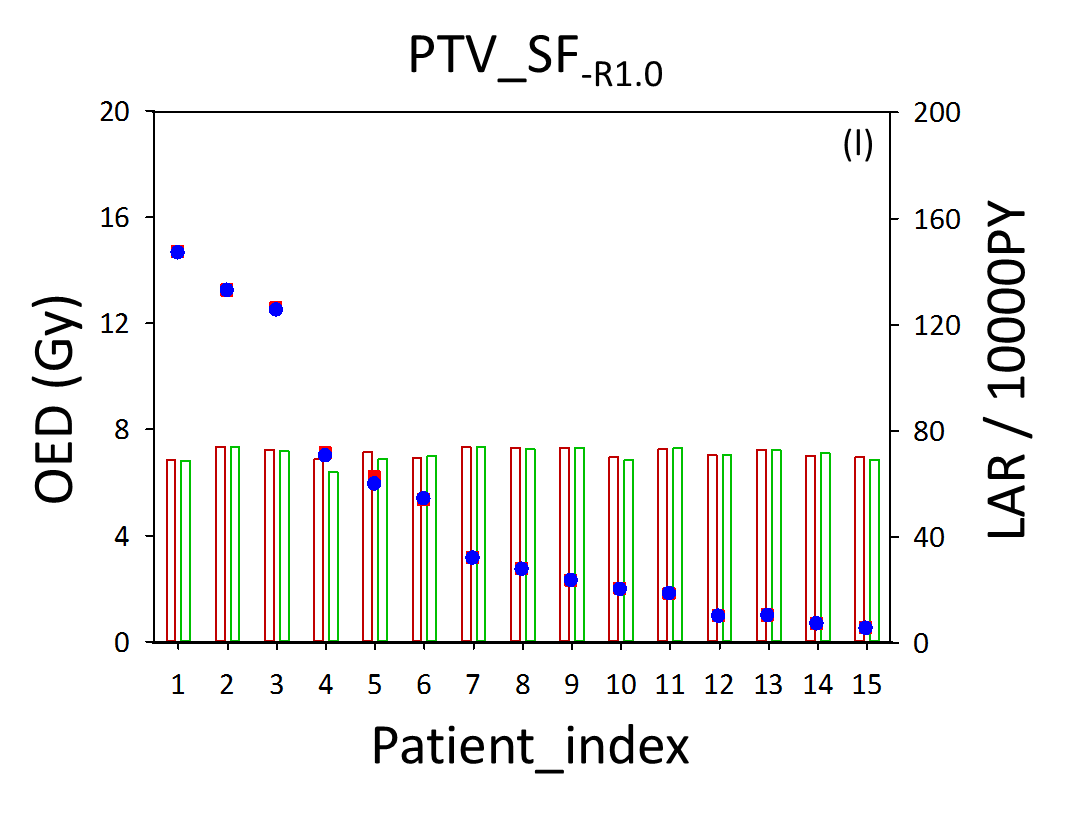


**Supplementary** **Figure S2.** OED/LAR of 15 patients using corresponding dose-response model stratified by the two techniques of MLC and IRIS. (a-d) for brain stem; (e-h) for soft tissue; (i-l) for PTV.

**Notes:** The EAR has units of excess cases per 10,000 person-years (PY)/Gy. Schneider dose-response model with repopulation/repair effects by using Eq.2 with a fixed limit of R; low repopulation (SL_-R0.1_) R = 0.1, intermediate repopulation (SI_-R0.5_) R = 0.5, full tissue recovery models (SF_-R1.0_) R = 1.0; The results show that the younger a patient is at the time of radiation treatment, the higher the LAR is for a future secondary cancer. Patients were ordered by age from young to old.

**Abbreviations:** MLC, multi-leaf collimator; IRIS, Iris collimator; Lnt, Linear-no-threshold dose response model; LinExp, linear-exponential dose response model; Plateau, Plateau dose response model; Full, Schneider parameterization dose-response model; PTV, Planning target volume.
